# Single-cell and isoform-specific translational profiling of the mouse brain

**DOI:** 10.1038/s41586-026-10118-1

**Published:** 2026-02-18

**Authors:** Samantha L. Sison, Federico Zampa, Eric R. Kofman, Su Yeun Choi, Pratibha Jagannatha, Grady G. Nguyen, Jack T. Naritomi, Asa Shin, Akanksha Khorgade, Wenhao Jin, Chun-Yuan Chen, David M. Sievert, Sourish Mukhopadhyay, Orel Mizrahi, Steven M. Blue, Ryan J. Marina, Dong Yang, Cailynn C. Wang, Zhengyuan Pang, Kristopher W. Brannan, Li Ye, Aziz M. Al’Khafaji, Gene W. Yeo, Giordano Lippi

**Affiliations:** 1 Department of Cellular and Molecular Medicine, University of California San Diego, La Jolla, CA USA; 2 Dorris Neuroscience Center, Scripps Research, La Jolla, CA USA; 3 Center for RNA Technologies and Therapeutics, University of California San Diego, La Jolla, CA USA; 4 Broad Institute of MIT and Harvard, Cambridge, MA USA; 5 Sanford Laboratories for Innovative Medicines, San Diego, CA USA; 6 Sanford Stem Cell Institute Innovation Center, University of California San Diego, La Jolla, CA USA; 7 Department of Biological Chemistry and Molecular Pharmacology, Harvard Medical School, Boston, MA USA; 8 Department of Chemistry, Scripps Research, La Jolla, CA USA; 9 Houston Methodist Research Institute, Houston, TX USA

**Keywords:** Cellular neuroscience, Molecular neuroscience, Translation, Transcriptomics, RNA splicing

## Abstract

The brain displays the richest repertoire of post-transcriptional mechanisms regulating mRNA translation^1–11^. Among these, alternative splicing has been shown to drive cell-type specificity and, when disrupted, is strongly linked to neurological disorders^12–17^. However, genome-wide measurements of mRNA translation with isoform sensitivity at single-cell resolution have not been achieved. To address this, we deployed Surveying Ribosomal Targets by APOBEC-Mediated Profiling (Ribo-STAMP) coupled with short-read and long-read single-cell RNA sequencing in the brain^18^. We generated the first isoform-sensitive single-cell translatomes of the mouse hippocampus at postnatal day 25, discovering cell-type-specific translation of 3,857 alternative transcripts across 1,641 genes and identifying isoforms of the same genes undergoing differential translation within and across 8 different cell types. We defined high and low translational states in CA1 and CA3 neurons, with synaptic and metabolic genes enriched in high states. We found that CA3 exhibited higher basal translation compared with CA1, as confirmed by metabolic labelling of newly synthesized proteins and immunohistochemistry of translational machinery components. This accessible platform will expand our understanding of how cell-type-specific and isoform-specific translation drives brain physiology and disease.

## Abstract (web summary)

Post-transcriptional regulation of mRNA translation was explored using Ribo-STAMP and single-cell RNA sequencing to reveal cell-type-specific and isoform-specific translation patterns across hippocampal neuronal and non-neuronal cell types, highlighting functional differences between CA1 and CA3.

## Main

Cell-type diversity and function in the brain are controlled by post-transcriptional regulatory mechanisms mediated by RNA-binding proteins (RBPs) and microRNAs (miRNAs)^1,6,7,10,19^. The brain exhibits a greater variation of alternative splicing, 3′ untranslated region (UTR) length and RNA modifications, all of which have roles in the translational control of mRNA to protein^2–5,8,9,11–16^. Among these mechanisms, alternative splicing stands out as an effective and pervasive source of transcriptome diversity. Nearly 95% of genes in humans and 63% in mice are alternatively spliced, which can lead to vastly different proteoforms and protein interactomes^20,21^. In the nervous system, isoform diversity varies across sex, developmental stages and brain regions^13,15,16^, and mis-splicing has strong links to neuropsychiatric disorders^12,17,22^. Isoform diversity underlies and defines neuronal cell-type specificity^13,15–17^. Despite this, the extent to which isoforms are differentially translated across cells of the brain is poorly understood.

The low correlation between RNA and protein abundance in the brain is well documented^23^. Genome-wide technologies, such as ribosome profiling (Ribo-seq) and polysome profiling, revealed patterns underlying mRNA translation and differential translation^24^ but are not readily available for low input material. Although platforms such as short-read single-cell RNA sequencing (scRNA-seq)^14,25,26^ are effective in characterizing gene expression, tools capable of simultaneously characterizing translation in several cell types at single-cell resolution are needed. Recent technological breakthroughs showed proof-of-concept single-cell translational profiling, down to the spatial level^27–29^. However, limitations remain (Supplementary Fig. 1), such as detecting isoform specificity. Long-read scRNA-seq revolutionized our understanding of isoform diversity but is not compatible with current translational profiling technologies^13,15,30^.

**Fig. 1:**
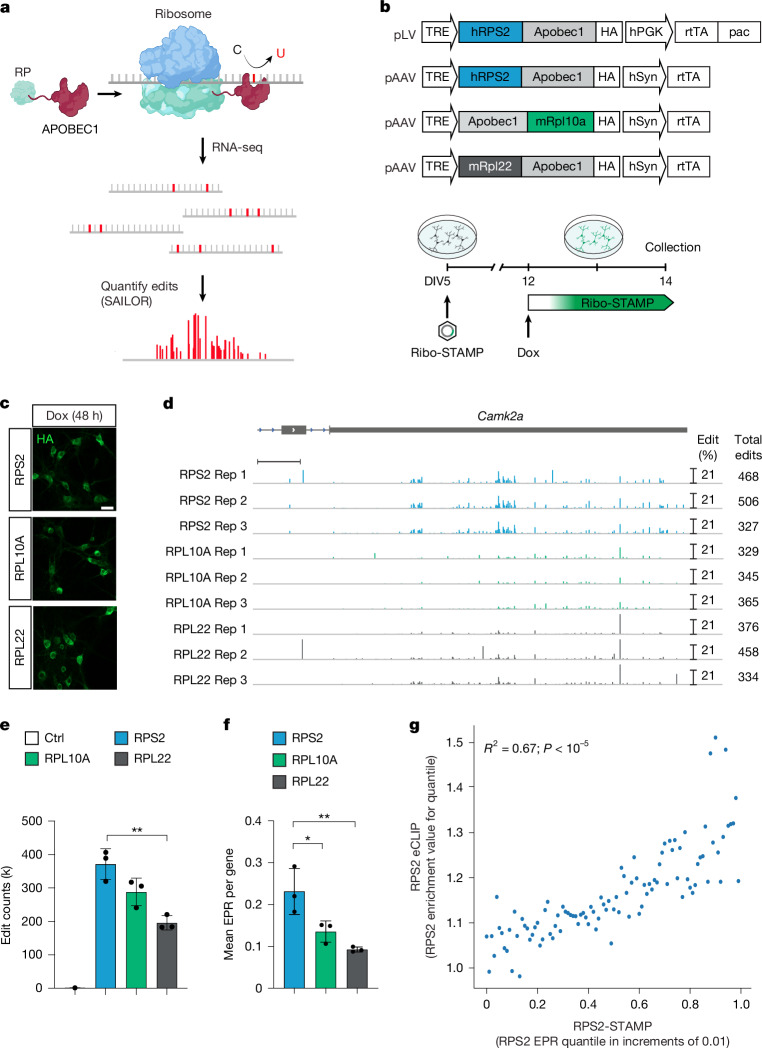
**Ribo-STAMP detects mRNA translation in primary neurons.** **a**, The Ribo-STAMP technology fuses rAPOBEC1 to a ribosomal protein (RP), inducing C-to-U edits at or near ribosome binding sites on mRNAs. Edits are detected by standard RNA-seq and quantified using the SAILOR analysis pipeline. **b**, Schematic of the original lentiviral (lentiviral plasmid, pLV) and neuron-specific AAV Ribo-STAMP expression vectors (pAAV, top). Schematic of the experimental paradigm for Ribo-STAMP expression in primary neurons (bottom). **c**, Detection of Ribo-STAMP variant expression with haemagglutinin (HA) tag immunocytochemistry. **d**, Integrative genome viewer (IGV) browser tracks showing edit peaks from the tested Ribo-STAMP variants on the *Camk2a* gene from biological replicates. Edit peaks are shown as percentage of edited Cs, and total *Camk2a* edit counts for each condition and replicate are reported on the right. **e**, Total edit counts deposited by the Ribo-STAMP variants and in uninfected neurons (control, Ctrl). **f**, Mean EPR per gene levels. **g**, Correlation between RPS2 eCLIP and RPS2-STAMP EPR; Pearson *R* (**e**,**f**). Bars represent mean ± s.d. from *n* = 3 biological replicates. Ctrl is from *n* = 2 biological replicates. One-way analysis of variance (ANOVA) followed by Tukey’s multiple comparison post hoc test. *P*_adjust_ = 0.003 (**e**). RPS2 versus RPL10A, *P*_adjust_ = 0.0361; RPS2 versus RPL22, *P*_adjust_ = 0.007 (**f**). *P < 0.05; **P < 0.01. DIV, day in vitro; hPGK, human phosphoglycerate kinase; hSyn, human synapsin promoter; pac, puromycin *N*-acetyl transferase; TRE, tetracycline-responsive promoter element. Scale bars, 20 μm (**c**), 500 bp (**d**). Illustrations in **a** and **b** were created in BioRender; Lippi, G. https://biorender.com/ri8rh7h (2025).

To overcome this, we applied Surveying Ribosomal Targets by APOBEC-Mediated Profiling (Ribo-STAMP)^18^ in the brain and coupled it with short-read and long-read RNA-seq to measure translation at single-cell and isoform resolution. Previously, we demonstrated that Ribo-STAMP is an efficient method for simultaneously measuring transcription and translation in single cells using the RNA editing enzyme APOBEC1 fused to human ribosomal protein S2 (RPS2), which deposits C-to-U edits on translating transcripts^18,31^ (Fig. 1a). Ribo-STAMP correlates with Ribo-seq and polysome profiling and detects Torin-1-induced translational inhibition and differential translation in cancer cells^18,31–33^. Additionally, Ribo-STAMP can be used for isoform-sensitive translational profiling using long-read bulk RNA-seq^34^. Taking advantage of the recent improvement of throughput with multiplexed arrays isoform sequencing (MAS-ISO-seq)^35^, we demonstrate that Ribo-STAMP is capable of isoform-sensitive translational profiling of cellular diversity and function across single cells in the brain.

## Ribo-STAMP detects translation in neurons

**Extended Data Fig. 1:**
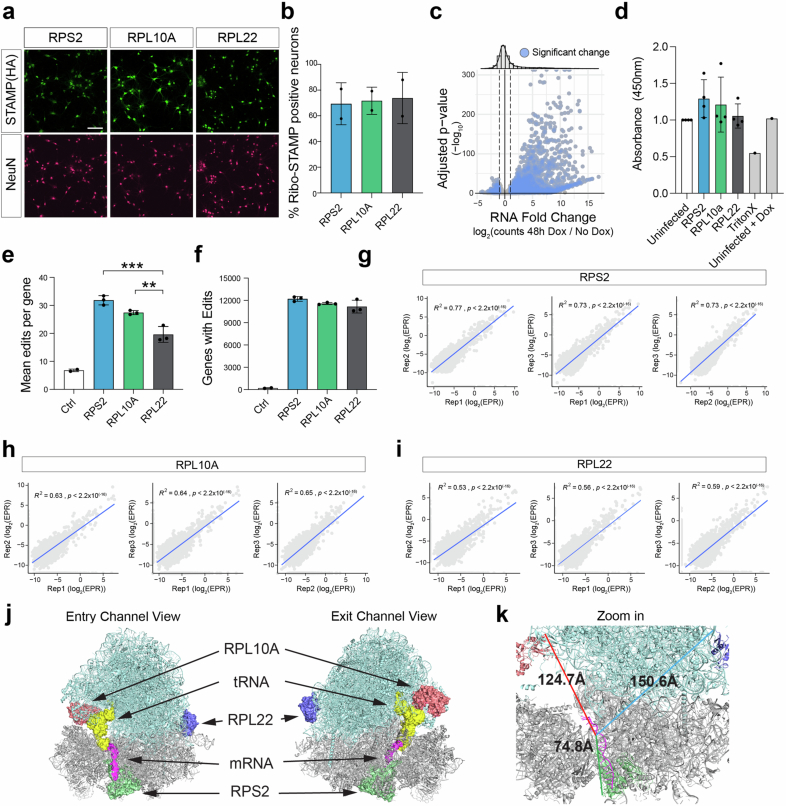
Comparison of Ribo-STAMP variants. **a**,**b**, Comparable infection rate of Ribo-STAMP variants quantified as percentage of NeuN positive neurons. Scale bar, 100 μm. **c**, Differential expression analysis between 48 h Dox-treated and non-treated neurons (significant RNA enrichment: log_2_FC < −1 or >1, Wald test, Benjamini–Hochberg correction, *P* < 0.01). We observed an increase in RNA levels for 2233 protein-coding genes and a decrease in RNA levels for 710 genes. **d**, Cell Counting Kit-8 assay measurement of mitochondrial activity. **e**, Mean number of edits per gene. **f**, Number of genes containing edits. **g-i**, Correlations of EPR between biological replicates for Ribo-STAMP variants; Pearson *R*. **j**, Positioning of RPL10A, tRNA, RPL22, mRNA and RPS2 within reconstructed mouse ribosome. **k**, Calculated distance between the APOBEC1-fused termini of RPS2, RPL10A, and RPL22 and the center of the RNA molecule. (**b**,**d**,**e**,**f**) Bars represent mean ± s.d., significant comparisons are shown from (**b**) *n* = 2, (**c**) *n* = 3, (**d**) *n* = 4 biological replicates, except *n* = 1 for TritonX and Uninfected + Dox, (**e**,**f**) *n* = 3 biological replicates except *n* = 2 for ctrl (**d**) One-way ANOVA followed by Dunnett’s multiple comparisons post-hoc test, comparison to “Uninfected” condition. (**e**,**f**) One-way ANOVA followed by Tukey’s multiple comparisons post-hoc test, **e**: RPS2 vs RPL22 *P*_*adjust *_ = 0.0006, RPL10A vs RPL22 *P*_*adjust *_ = 0.0063; **P < 0.01, ***P < 0.001.

To optimize Ribo-STAMP for expression in brain cells in vivo, we generated an adeno-associated virus (AAV) Tet-On Ribo-STAMP expression vector with a synapsin promoter driving reverse tetracycline-controlled transactivator (rtTA) for neuronal expression. In addition to the hRPS2–APOBEC1 design, we evaluated two Ribo-STAMP variants on the basis of the translating ribosome affinity purification (TRAP) and RiboTag systems, in which APOBEC1 was fused to the mouse Rpl10a and Rpl22 open reading frames (ORFs), respectively (Fig. 1b). After 48-h induction with doxycycline (Dox), all constructs were robustly expressed in primary mouse cortical neurons (Fig. 1c and Extended Data Fig. 1a,b). Following short-read bulk RNA-seq, we quantified edits with the SAILOR algorithm. C-to-U edits were deposited by all Ribo-STAMP variants, as illustrated by the neuronal marker gene *Camk2a* (Fig. 1d). As edits in the coding sequence (CDS) might affect protein synthesis, we assessed whether Ribo-STAMP caused editing-induced toxicity. Differential expression analysis between non-treated and Dox-treated neurons showed a subset of differentially expressed genes (DEGs) (Extended Data Fig. 1c and Supplementary Table 1). However, the DEGs were not enriched for the ‘response to stress’ Gene Ontology term, suggesting that neurons are not experiencing toxicity. In addition to Ribo-STAMP expression, Dox alone is known to affect gene expression^36^. Therefore, Dox-induced DEGs were removed from downstream bulk RNA-seq analysis. After 72 h of Ribo-STAMP expression, cell viability was not impaired (Extended Data Fig. 1d). We concluded that induction of neuronal-specific Ribo-STAMP expression for up to 72 h does not cause adverse effects in neurons.

To select the most efficient Ribo-STAMP variant, we compared editing performance. Although all variants robustly deposited edits, RPS2-STAMP deposited 1.3-fold more edits than Rpl10a–STAMP and 1.9-fold more than Rpl22–STAMP (Extended Data Fig. 1e,f). To better evaluate translational efficiency with Ribo-STAMP across samples, similarly to the Ribo-seq translational efficiency metric of ribosome-associated reads divided by total reads, we calculated the number of edits per number of reads (EPR). Unlike in Ribo-seq, Ribo-STAMP edits are made on the same RNA molecules in the same cells, constructed and sequenced in the same library, allowing for a more veritable, internally normalized metric of translational efficiency. RPS2-STAMP displayed the highest EPR among the different Ribo-STAMP variants (Fig. 1f), in line with the overall edit deposition (Fig. 1e and Extended Data Fig. 1e,f). Although all variants showed positive EPR correlation between technical replicates, RPS2-STAMP exhibited the highest reproducibility (Extended Data Fig. 1g–i), possibly because of higher edit deposition. In a simulated mouse ribosome structure, compared with RPL10A and RPL22, RPS2 is in closer proximity to the centre of the RNA molecule (Extended Data Fig. 1j,k), potentially explaining the better edit deposition.

To determine whether RPS2-STAMP accurately reports ribosome occupancy in neurons, we benchmarked it against enhanced crosslinking and immunoprecipitation (eCLIP) of ribosomal proteins, a technique that captures ribosome interaction sites akin to ribosome profiling and has been previously used as a direct comparison for Ribo-STAMP^18,37^. Endogenous RPS2 eCLIP in primary neurons showed that it is positively correlated with RPS2-STAMP (Fig. 1g). In summary, we deemed RPS2-STAMP (hereafter Ribo-STAMP) as appropriate for translational measurements in neurons.

## Ribo-STAMP detects translation dynamics

**Fig. 2:**
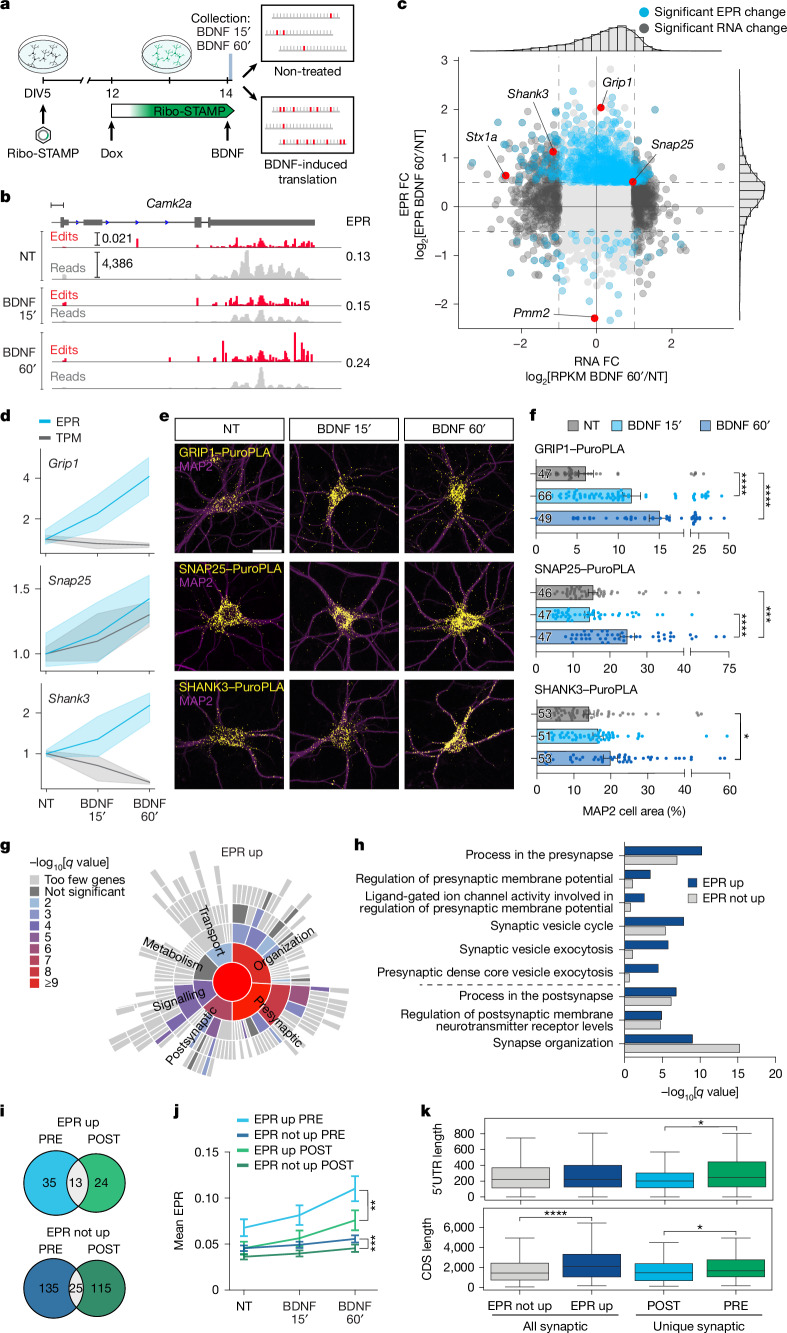
**Ribo-STAMP detects BDNF-induced translational dynamics.** **a**, Experimental paradigm. **b**, IGV browser tracks showing counts and edit peaks for *Camk2a* in non-treated and BDNF-treated primary neurons, with mean EPR levels (right). Edit peaks shown as percentage of edited Cs. **c**, EPR and RNA differential expression analysis comparing 60′ BDNF-induced and no treatment. Significant EPR change: two-sided *t*-test; *P* < 0.05; log_2_FC > 0.5 or < −0.5. Significant RNA change: Wald test; Benjamini–Hochberg correction; *P* < 0.01; log_2_FC > 1 or < −1. Validation candidates highlighted in red. **d**, EPR and TPM for candidates normalized to no treatment (NT) = 1. The shaded area is s.e.m. **e**, Puro-PLA representative images. **f**, Quantification of Puro-PLA positive area from **e** as percentage of MAP2 cell area. Cell numbers (in columns) over *n* = 3 biologically independent experiments. **g**, SynGO analysis of BDNF ‘EPR up’ (log_2_FC > 0.5; *P* < 0.05; two-sided *t*-test) genes. One-sided Fisher’s exact test and Benjamini–Hochberg correction. **h**, Comparison of example presynaptic and postsynaptic term enrichment from ‘EPR up’ and ‘EPR not up’ SynGO analyses. **i**, Number of unique presynaptic and postsynaptic genes found in ‘EPR up’ and ‘EPR not up’, and number of shared genes. **j**, EPR levels of genes in **i**. **k**, Comparison of CDS and 5′ UTR length among the gene lists in **h** and **i** combined (‘EPR up’ + ‘EPR not up’). All synaptic ‘EPR not up’ *n* = 818, all synaptic ‘EPR up’ *n* = 192, unique postsynaptic *n* = 139 and unique presynaptic *n* = 170 genes. Box plots show the interquartile range, with the line inside marking the median. Whiskers extend up to 1.5 times the interquartile range from the lower and upper quartiles. Bars represent mean ± s.e.m. (**f**,**j**). Kruskal–Wallis test followed by Tukey’s multiple comparison post hoc test (**f**); GRIP1, *P*_adjust_ < 0.0001; SNAP25, no treatment versus BDNF 60′ *P*_adjust_ = 0.0003; BDNF 15′ versus BDNF 60′ *P*_adjust_ < 0.0001; SHANK3, *P*_adjust_ = 0.0369. Two-sided Mann–Whitney *U*-test (**j**,**k**). Top, *P* = 0.002; bottom, *P* = 0.0008 (**j**). Top, *P* = 0.02414; bottom left, *P* = 1.322 × 10^−6^ (**k**). *P < 0.05; **P < 0.01; ***P < 0.001; ****P < 0.0001. Scale bars, 500 bp (**b**), 20 μm (**e**).

**Extended Data Fig. 2:**
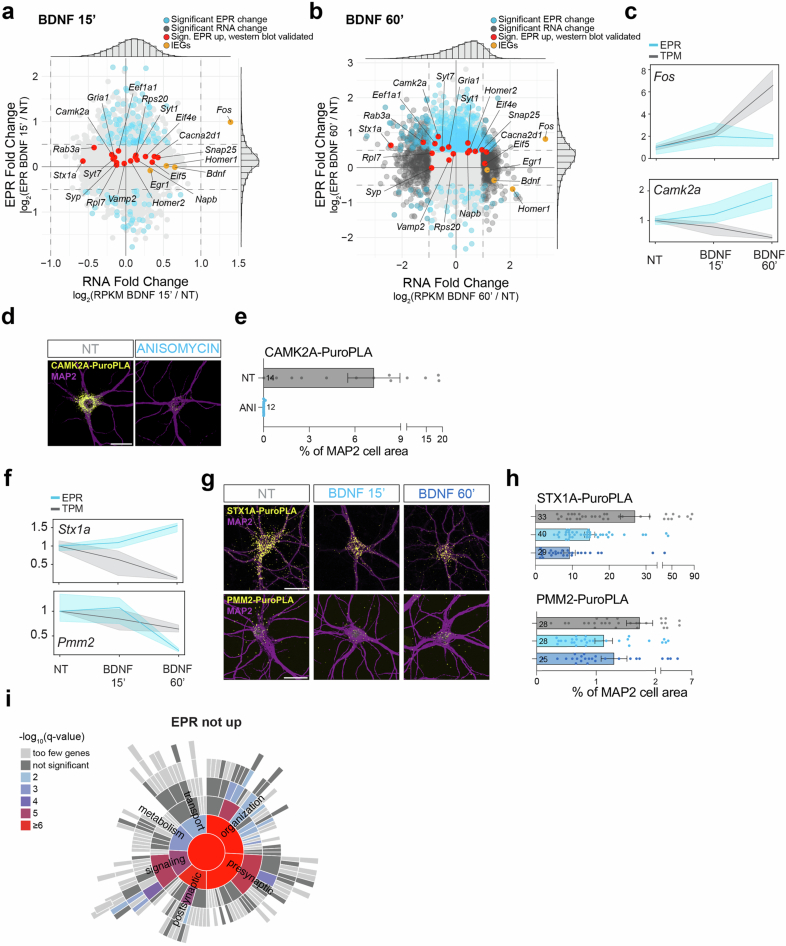
Validations of BDNF-induced EPR and RNA changes. **a**,**b**, Differential EPR and RNA analysis between 15′ BDNF-treated and NT neurons (**a**) and between 60′ BDNF-treated and NT neurons (**b**, same graph as in Fig. 2c). Significant EPR change: log_2_FC = >0.5 or <−0.5, *P* < 0.05. Significant RNA change: log_2_FC = >1 or <−1, Two-sided Wald test, Benjamini–Hochberg correction, *P* < 0.01. Literature-validated increase for immediate early genes (IEGs), highlighted in orange, and translationally induced genes, highlighted in red. **c**, EPR and TPM values for *Fos* and *Camk2a*. **d**, CAMK2A Puro-PLA representative images of non-treated and anysomycin-treated neurons. **e**, Quantification of Puro-PLA positive area in (**d**) as percentage of MAP2 cell area. Cells numbers indicated in columns from *n* = 2 biologically independent experiment. **f**, EPR and TPM values for *Stx1a* and *Pmm2*. **g**, Puro-PLA representative images for indicated candidates from (**f**). **h**, Quantification of Puro-PLA positive area from (**g**) as percentage of MAP2 cell area. Cells numbers indicated in columns from *n* = 2 biologically independent experiment. **i**, SynGO analysis of ‘EPR not up’ genes. One-sided Fisher exact test, Benjamini–Hochberg correction. (**d**,**g**) Scale bar, 20 μm. (**e,h**) Bars represent mean ± s.e.m.

To evaluate the sensitivity of Ribo-STAMP in detecting rapid translational changes (minutes to hours), we treated neurons with brain-derived neurotrophic factor (BDNF) to elicit protein synthesis and synaptic plasticity. We induced Ribo-STAMP expression in primary cortical neurons for 48 h with Dox and then treated with BDNF (100 ng ml^−1^) for 15′ or 60′ (Fig. 2a). Transcriptome-wide EPR values were obtained from bulk RNA-seq. We validated that *Camk2a*, a known BDNF target, increased editing after BDNF treatment (Fig. 2b). We measured differential RNA and EPR changes after BDNF treatment compared with no treatment (Fig. 2c). First, we confirmed the transcriptional induction of immediate early genes (IEGs), known to be BDNF-induced and activity-induced (Extended Data Fig. 2a–c). Next, we identified genes exhibiting differential EPR between BDNF-treated and no treatment conditions. In total, 878 genes showed a significant change in EPR, with 94% of these genes having increased EPR. Among the EPR-increased genes after 60′ BDNF, we found previously validated transcripts (Extended Data Fig. 2b,c and Supplementary Table 2). The EPR values of *Fos* and other IEGs were not significantly upregulated, suggesting that their BDNF-induced increase in protein levels is driven by transcriptional induction instead. Moreover, 86% of genes displaying significant EPR changes after 60′ BDNF were mildly induced at the transcriptional level (RNA log_2_ fold change (FC) between −1 and 1), suggesting that Ribo-STAMP is sensitive to identifying fast activity-induced translation that is not driven by large transcriptional changes.

To orthogonally validate the BDNF-induced translational changes detected with Ribo-STAMP, we used the puromycin proximity ligation assay (Puro-PLA), which visualizes newly synthesized proteins. As a control, neurons treated with the translational inhibitor anisomycin displayed a significant reduction in newly synthesized CAMK2A Puro-PLA puncta (Extended Data Fig. 2d,e). We validated a subset of genes identified (from Fig. 2c) that showed different EPR and RNA induction profiles after 60′ BDNF (Fig. 2d–f and Extended Data Fig. 2f–h). Change in EPR correctly predicted newly synthesized protein changes after BDNF treatment for all candidates in which RNA levels were modestly changing (GRIP1, SNAP25 and PMM2).

BDNF induces local presynaptic and postsynaptic translation required for synaptic plasticity^3,38^. However, the composition of the BDNF-induced translatome within synaptic compartments remains elusive. We focused on genes for which EPR was upregulated after 60′ BDNF treatment (‘EPR up’) versus the rest of the genes (‘EPR not up’). Using SynGO, an expert-curated dataset of synaptic genes, we observed that the ‘EPR up’ genes were enriched for presynaptic terms compared with ‘EPR not up’ genes. Postsynaptic and synapse organization terms showed overall similar enrichment between ‘EPR up’ and ‘EPR not up’ or enrichment in ‘EPR not up’ genes (Fig. 2g,h, Extended Data Fig. 2i and Supplementary Table 3).

To better understand compartment-specific translational regulation, we extracted the list of SynGO genes exclusively present in the presynaptic or postsynaptic datasets (‘PRE’ and ‘POST’, respectively; Fig. 2i and Supplementary Table 3). The ‘PRE’ genes showed higher basal and BDNF-induced EPR levels when compared with the ‘POST’ genes (Fig. 2j); however, BDNF-induced EPR increases for ‘PRE’ and ‘POST’ were comparable. To explain the differences in basal levels, we analysed gene features. We found that ‘EPR up’ genes (all genes) contain longer CDS compared with ‘EPR not up’ (Fig. 2k). CDS length negatively correlates with translational rates and ribosome density^39,40^. This could be a feature of genes that are lowly translated at baseline but increase translation after BDNF. Further, ‘PRE’ genes display longer CDS and 5′ UTR compared with ‘POST’ genes. Long 5′ UTRs are a feature of transcripts enriched in specialized neuronal compartments and enable complex translational regulation. Together, these results demonstrated that Ribo-STAMP effectively maps stimulus-induced translational changes of presynaptic and postsynaptic genes within a temporal window relevant for neuronal plasticity.

## In vivo single-cell translational profiling

**Fig. 3:**
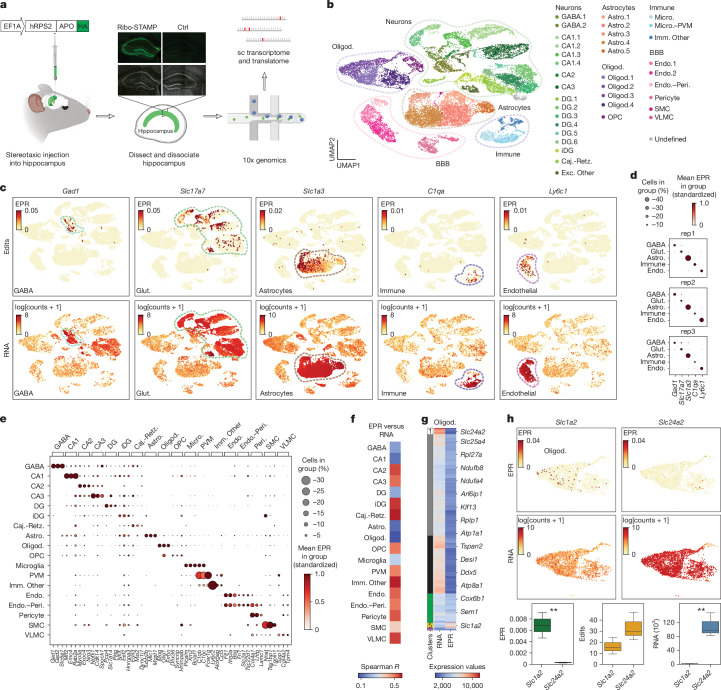
**Ribo-STAMP enables single-cell translational profiling of the mouse hippocampus.** **a**, Experimental design schematic and Ribo-STAMP HA tag immunohistochemistry (IHC). Anti-HA (green) and DAPI (grey). **b**, Uniform manifold approximation and projection (UMAP) of cells labelled by cell assignments. **c**, UMAPs of edits (EPR) and RNA (log[counts + 1]) of known marker genes from left to right. **d**, Mean EPR expression of known marker genes in the three individual replicates. **e**, Top 3 ranked genes per cell type by EPR. **f**, Correlations of pseudobulk EPR versus RNA for each cell type; Spearman’s *R*. **g**, Gene clustering of pseudobulk EPR and RNA in oligodendrocytes; *n* = 431 genes are shown. **h**, Example genes *Slc1a2* (from cluster 1) and *Slc24a2* (from cluster 5), showing opposite EPR and RNA expression. UMAPs (top) showing EPR and RNA (log[counts + 1]) and mean EPR, edits and RNA reads quantified per animal (bottom). Two-sided *t*-test; EPR *P* = 7.075 × 10^−3^; RNA *P* = 5.729 × 10^−3^; **P < 0.01; *n* = 3 mice. Box plots show the interquartile range, with the line inside marking the median. Whiskers extend up to 1.5 times the interquartile range from the lower and upper quartiles. Dot-plot colours represent mean standardized EPR by row; size of bubble represents percentage of cells in group (**d**,**e**). UMAPs: UMAP1 versus UMAP2 (**b**,**c**,**h**). Astro., astrocytes; BBB, blood–brain barrier; Caj.-Retz., Cajal–Retzius; DG, dentate gyrus; Endo., endothelial cells; Endo.–Peri., endothelial cells–pericytes; Exc. Other, excitatory neurons unassigned; GABA, GABAergic neurons; Glut., glutamate; iDG, immature dentate gyrus; Imm. Other, immunity cells, other; Micro., microglia; Oligod., oligodendrocytes; OPC, oligodendrocyte precursor cells; Peri., pericytes; PVM, perivascular macrophages; SMC, smooth muscle cells; VLMC, vascular leptomeningeal cells. Illustrations in **a** were created in BioRender; Lippi, G. https://biorender.com/cyjsgws (2025).

**Extended Data Fig. 3:**
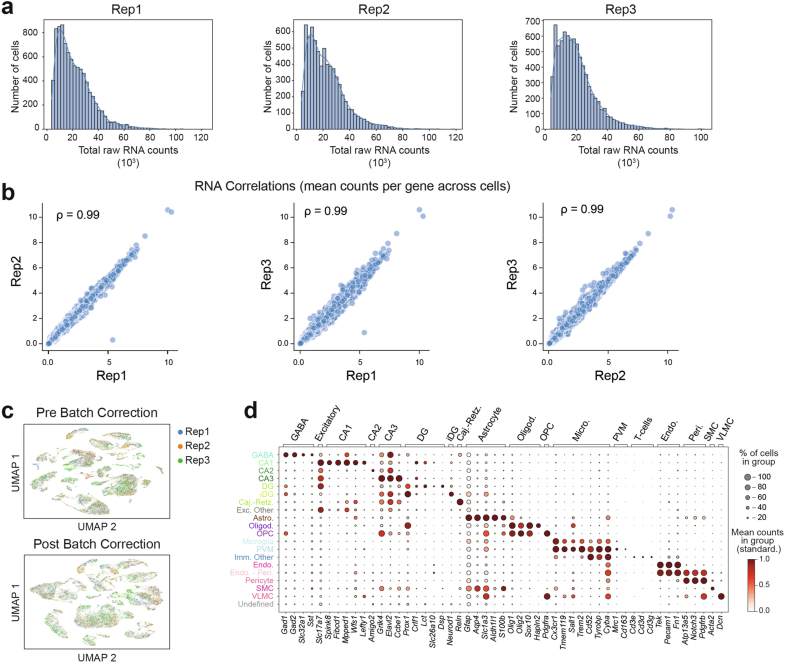
Cell type-specific expression in the mouse hippocampus. **a**, Distribution of cells by raw counts for the three replicates. **b**, Scatter plot of RNA Counts Correlations across replicates; Spearman R. **c**, UMAPs of RNA for the combined sc dataset prior and after batch correction. **d**, Dotplot used for cell assignments in Fig. 3b. Colors represent mean log(counts+1) standardized by row, size of bubble represent % of cells in group. (**c**) UMAPs: UMAP1 vs UMAP2. (**d**) GABA, GABAergic neurons; DG, dentate gyrus; iDG, DG immature; Caj.-Retz., Cajal Retzius; Astro., astrocytes; Oligod., oligodendrocytes; OPC, oligodendrocyte precursor cells; Micro., microglia; PVM, perivascular macrophages; Imm. Other, Immunitary cells, other; Endo., endothelial cells; Endo.-Peri., endothelial cells- pericytes; Peri., pericytes; SMC, smooth muscle cells; VLMC vascular leptomeningeal cells.

With the goal of identifying translational determinants of cell types, states and functions in the brain, we performed 10x Genomics-based scRNA-seq coupled with Ribo-STAMP in the mouse hippocampus, a brain region essential for learning and memory. To simultaneously map the translatome of all cell types, we transduced a constitutive AAV RPS2-STAMP construct and saw robust expression after 72 h (Fig. 3a). We dissociated cells from three independent mice and performed short-read scRNA-seq, which was highly correlated (Extended Data Fig. 3a,b). After batch correction, we annotated known hippocampal cell types on the basis of RNA expression (Extended Data Fig. 3c,d), including subclasses of neurons, glia and cell types of the blood–brain barrier (Fig. 3b and Extended Data Fig. 3d).

**Extended Data Fig. 4:**
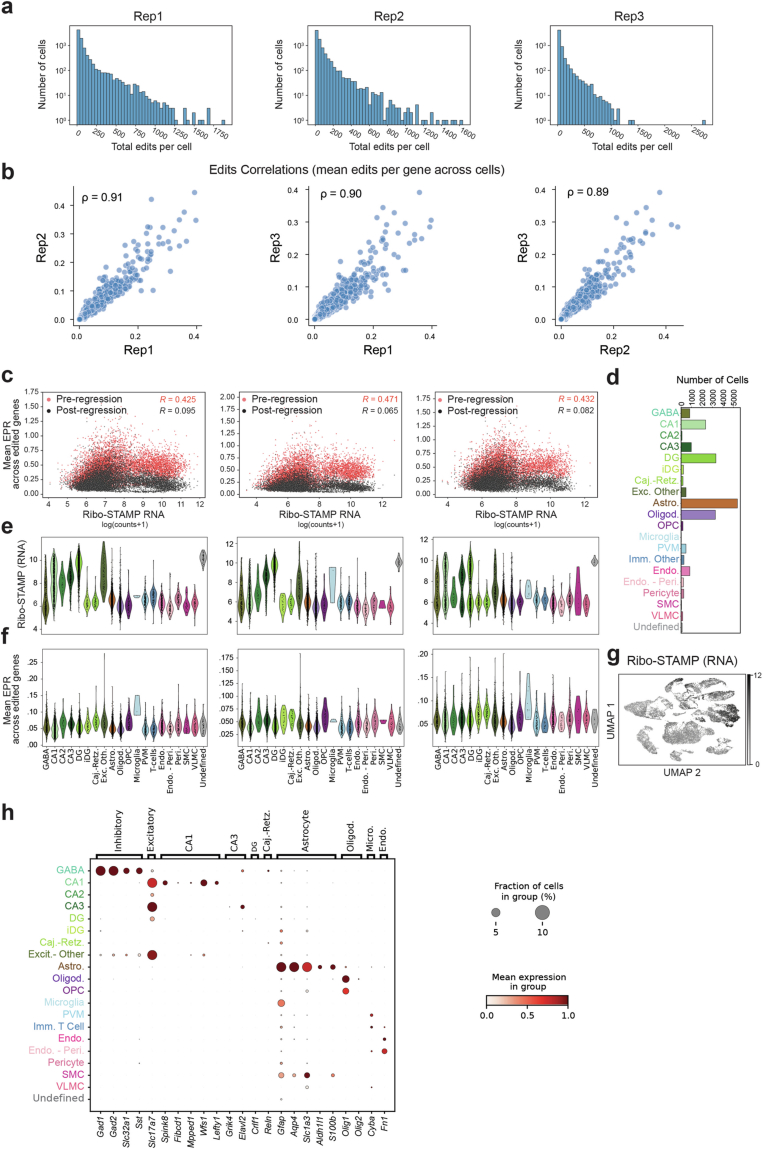
Cell type-specific editing in the mouse hippocampus. **a**, Distribution of cells by Edits for the three replicates. **b**, Scatter plot of edits Spearman correlations across replicates. **c**, Scatter plot of EPR by Ribo-STAMP RNA expression log(counts+1) before and after performing regression to normalize edits for Ribo-STAMP expression. Red, original EPR values; Black, post-regression normalized EPR values. Pre- and Post Spearman correlation *R* values are shown. **d**, Number of cells retained after filtering for the analysis. **e**, Ribo-STAMP RNA expression across cell types and replicates. **f**, Mean EPR per cell across cell types and replicates. **g**, UMAP of Ribo-STAMP construct RNA expression by log(counts+1) across all cells. **h**, Dotplot of marker genes from Extended Data Fig. 4d by EPR for genes that had edits. Colors represent mean EPR standardized by row, size of bubble represent % of cells in group. (**g**) UMAPs: UMAP1 vs UMAP2. (**d**,**e**,**f**,**h**) GABA, GABAergic neurons; DG, dentate gyrus; iDG, DG immature; Caj.-Retz., Cajal Retzius; Astro., astrocytes; Oligod., oligodendrocytes; OPC, oligodendrocyte precursor cells; Micro., microglia; PVM, perivascular macrophages; Imm. Other, Immunitary cells, other; Endo., endothelial cells; Endo.-Peri., endothelial cells- pericytes; Peri., pericytes; SMC, smooth muscle cells; VLMC vascular leptomeningeal cells.

**Extended Data Fig. 5:**
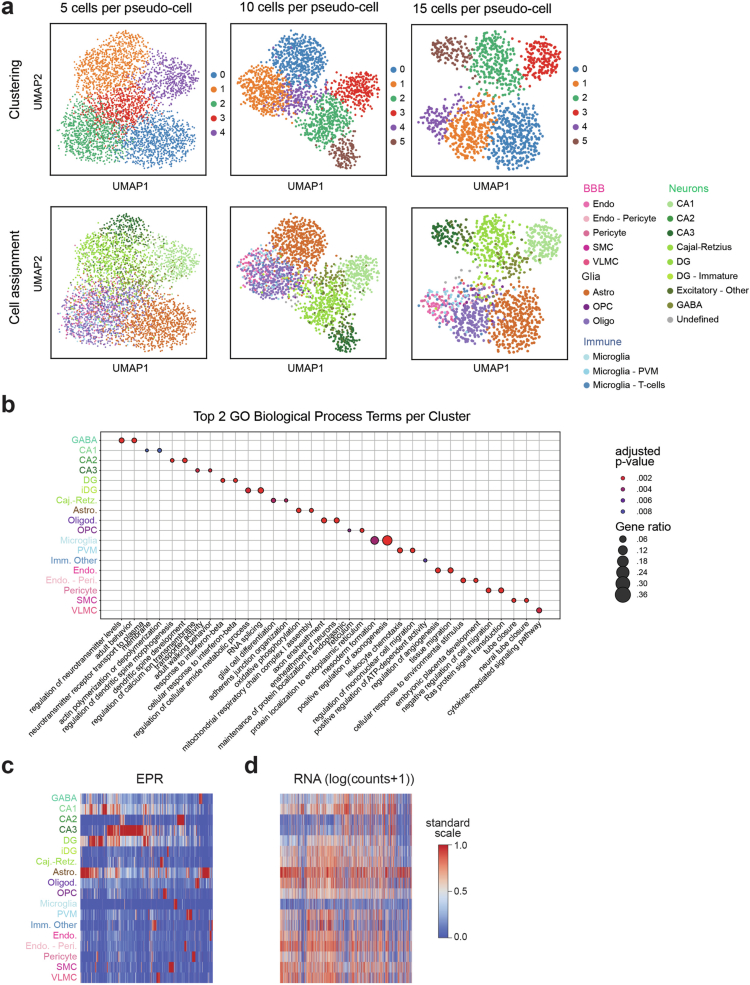
Clustering by EPR and cell type-specific GO terms and EPR and RNA expression clusters. **a**, EPR clustering shown using 5, 10, or 15 cells per pseudo-cell (see Methods). EPR-based leiden clusters (left) and original RNA cell assignments (right). **b**, GO analysis of biological process for top 100 EPR genes for each cell type. Hypergeometric test, Benjamini–Hochberg correction. **c**,**d**, Pseudo-bulk edits (EPR) by gene showing cell type-specific editing (**c**) and pseudo-bulk RNA (log(counts+1) by gene in the same order as EPR plot (**d**). Both plots show standardized EPR or log(counts+1) by row. *n* =1940 genes are shown.

We developed a computational workflow embodied in the Multi-Core Algorithm for Rapid Identification of Nucleotide Edits (MARINE) software repository (available at https://github.com/YeoLab/MARINE) to accurately and rapidly identify C-to-U edits at single-cell resolution. MARINE is a memory-efficient and ultra-fast Python package that rapidly identifies barcode-specific edits de novo, operating with a time complexity of O(*n*), where *n* is the number of edited reads, while memory usage is capped at a constant O(*c*) on the basis of genomic interval size used for edit-counting parallelization and on available resources. MARINE has superior performance for single-cell edit calls (with detailed comparisons to benchmarks in Supplementary Fig. 2). We removed edits overlapping with annotated single-nucleotide polymorphisms (SNPs). We calculated per-cell EPR values as total C-to-U edits divided by the total reads for each cell. Edit values between samples were highly correlated (Extended Data Fig. 4a,b). Because we observed a significant correlation between Ribo-STAMP expression and mean EPR for each sample, we performed linear regression to normalize edits for Ribo-STAMP expression (Extended Data Fig. 4c). We removed low-quality cells with fewer than five edited genes. This resulted in a dataset of 19,610 cells robustly expressing Ribo-STAMP (Extended Data Fig. 4e–g). We then evaluated mean EPR (Extended Data Fig. 4f) and found that EPR levels across cell types did not reflect Ribo-STAMP expression. To validate that Ribo-STAMP was accurately reporting cell-type-specific translation, we measured edit deposition on markers used for RNA cell-type assignments (Extended Data Fig. 4h) and known protein markers, including *Gad1* for inhibitory neurons, *Slc17a7* for excitatory neurons, *Slc1a3* for astrocytes, *C1qa* for immune cells and *Ly6c1* for endothelial cells (Fig. 3c). Indeed, these markers were selectively edited in the expected cell types across the three replicates (Fig. 3d). We clustered cells by EPR and found that clusters matched RNA cell assignments for cell types with the most numbers of cells, further validating that edits were deposited on cell-type relevant genes (Extended Data Fig. 5a). To identify translation-specific marker genes, we ranked genes for differential EPR in each cell type (Fig. 3e). This confirmed that all cell types translate genes linked to their function at high levels (for example, *Gad1*, which is important for GABA metabolism). Gene Ontology enrichment analysis confirmed that the top 100 genes differentially translated are related to known biological function of the cell type (for example, ‘axon ensheathment’ for oligodendrocytes; Extended Data Fig. 5b and Supplementary Table 4).

To evaluate the relationship between transcription and translation across cell types, we clustered genes by EPR. We found that CA3 had the most genes clustered by cell type. To understand if these patterns were reflected at the transcriptional level, we plotted the RNA counts of the same genes ordered by EPR (Extended Data Fig. 5c,d). We found that RNA counts did not show a clear pattern reflecting edits. Correlations between EPR and RNA for each cell type showed variability across cell types, with oligodendrocytes exhibiting the lowest correlation (Fig. 3f), validating the RIBOmap results^27^. We further explored the transcription–translation relationship in oligodendrocytes through clustering genes by EPR and RNA (Fig. 3g). We identified eight clusters of genes that had differential EPR and RNA, with cluster 1 exhibiting high RNA and low EPR and cluster 5 displaying low RNA and high EPR. We highlight two genes, *Slc1a2* (from cluster 5) and *Slc24a2* (from cluster 1) (Fig. 3h), demonstrating the ability of Ribo-STAMP to reveal unique transcriptional and translational programs in brain cells.

## Translational cell states in brain cells

**Fig. 4:**
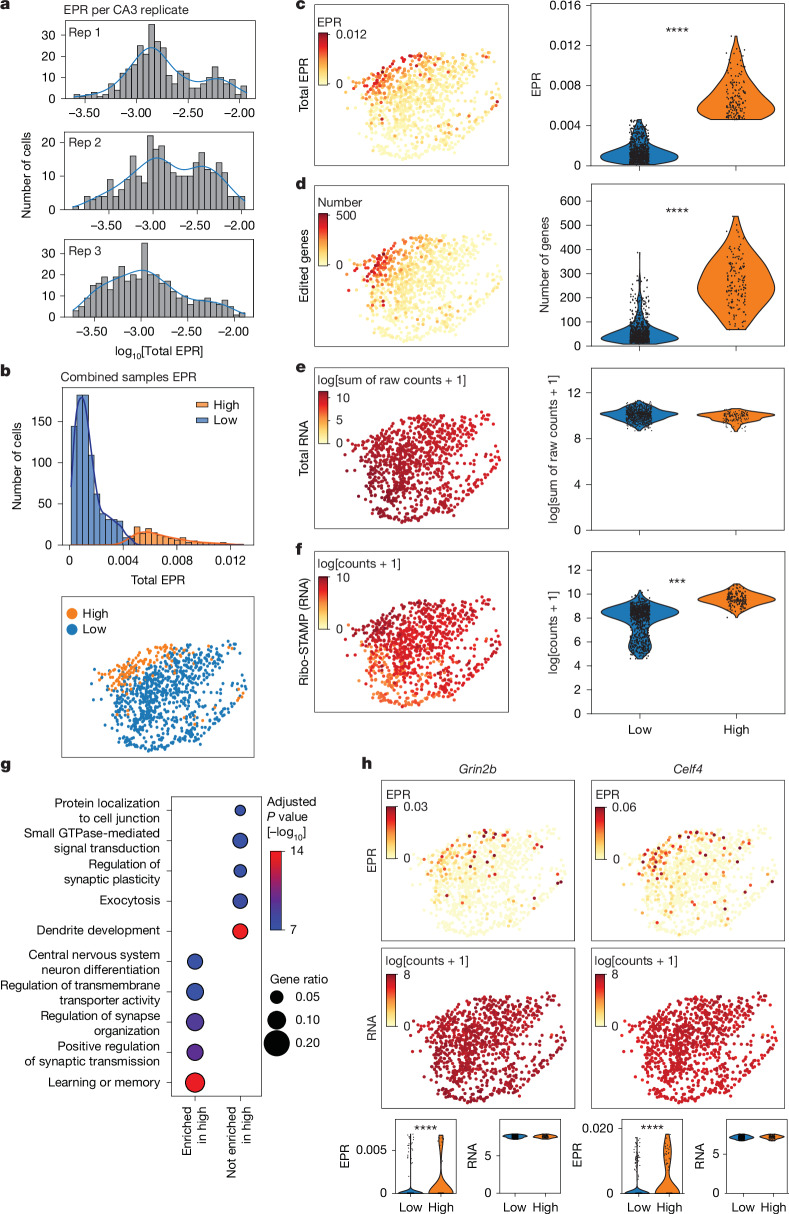
**Discovery of translational cell states in mouse brain cells.** **a**, Distribution of CA3 cells by EPR for individual replicates. **b**, Top, combined EPR distribution and clustering; bottom, UMAP visualization of CA3 cells annotated with high and low translation states from clustering. **c**–**f**, Total EPR (*P* = 1.38 × 10^−5^) (**c**), number of edited genes (*P* = 1.08 × 10^−5^) (**d**), total RNA expression (log of the sum of raw counts + 1) (**e**) and Ribo-STAMP RNA expression (*P* = 4.00 × 10^−4^) (**f**) for high and low translation states. UMAPs (left) and quantifications (right; two-sided Student’s *t*-test; mean values across *n* = 3 animals). **g**, Gene Ontology analysis of EPR-enriched genes in the CA3 high translation cluster versus non-enriched genes. Enriched in high, *n* = 279; not enriched in high, *n* = 775. Hypergeometric test; Benjamini–Hochberg correction. **h**, Expression of example genes associated with synaptic terms in **g**. *Grin2b* and *Celf4* shown as EPR and RNA (log[counts + 1]). Top, UMAPs; bottom, quantifications. *Grin2b* EPR *P*_adjust_ = 2.52 × 10^−13^; Celf4 EPR *P*_adjust_ = 2.68 × 10^−20^; two-sided Mann–Whitney *U*-test; Benjamini–Hochberg correction (**c**–**f**,**h**); UMAPs, UMAP1 versus UMAP2. ***P < 0.001; ****P < 0.0001.

**Extended Data Fig. 6:**
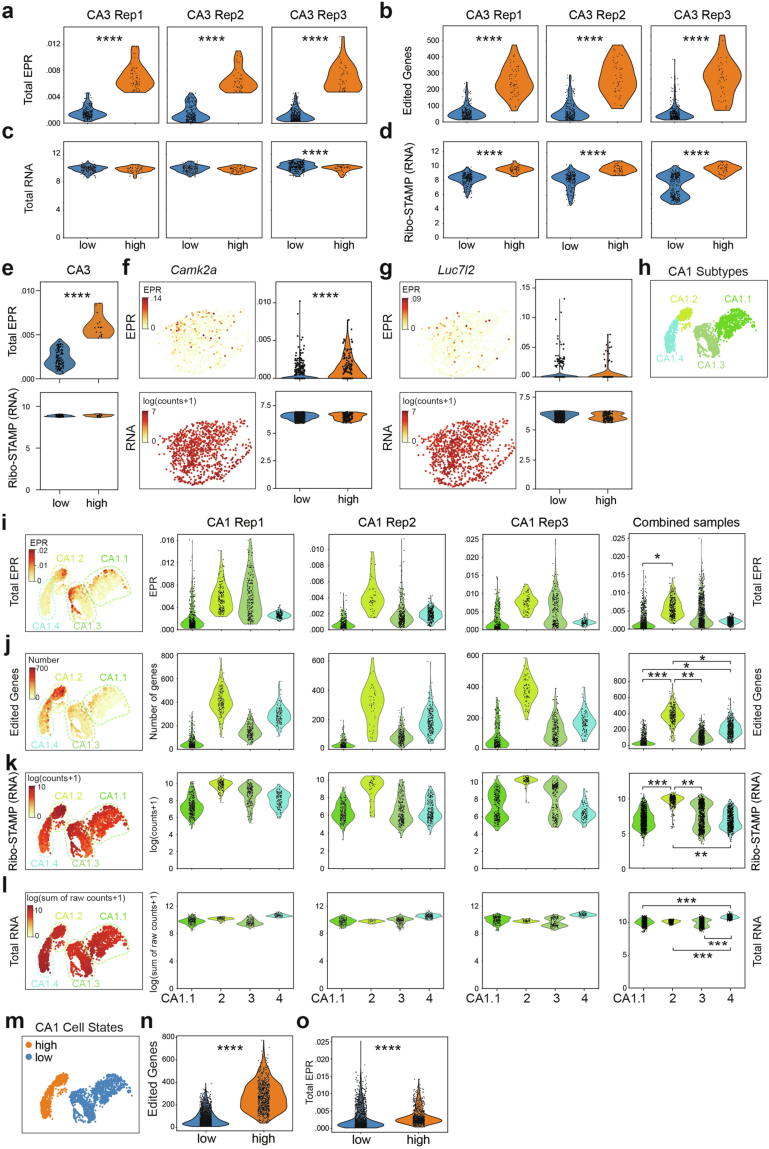
Detection of translational states in CA3 and CA1 neurons. **a-d**, Quantifications in (Fig. 4c–f) shown as individual replicates. **e**, Total EPR and Ribo-STAMP RNA quantifications from cells within Ribo-STAMP 0.65 and <0.8 quantiles. **f**,**g**, Expression of example genes from GO terms analysis in (Fig. 4g), enriched in CA3 ‘high’ translation cluster *Camk2a* (**f**), or not enriched, *Luc7l2* (**g**), shown as EPR and RNA (log(counts+1)) UMAPs (top) and quantifications (bottom). **h**, CA1 transcriptional clusters as identified in (Fig. 3b). **i-l**, Total EPR, number of edited genes, Ribo-STAMP RNA expression, total RNA expression for the four CA1 clusters ‘shown as UMAPs with combined cells (left column) and quantifications from individual or combined replicates (middle-right columns). **m**, Clustering of CA1 neurons into ‘high’ and ‘low’ translational states. **n-o**, Number of edited genes and total EPR for CA1 ‘high’ and ‘low’ translational states cluster. (**f**-**m**) UMAPs: UMAP1 vs UMAP2. (**a-g**,**n-o**) Two-sided Mann-Whitney U test; **a-f** and **n-o**: all *P* < 10^−5^ except **c**: Total RNA Rep3: *P* = 0.00017; (**i,j,k,l**) One-way ANOVA followed by Tukey’s post-hoc comparisons; **i**: CA1.1 vs CA1.2 *P*_*adjust*_ = 0.0341; **j**: CA1.1 vs CA1.2 *P*_*adjust*_ = 0.0002, CA1.1 vs CA1.4 *P*_*adjust*_ = 0.0127, CA1.2 vs CA1.3 *P*_*adjust*_ = 0.0012, CA1.2 vs CA1.4 *P*_*adjust*_ = 0.0257; **k**: CA1.1 vs CA1.2 *P*_*adjust*_ = 0.0009, CA1.2 vs CA1.3 *P*_*adjust*_ = 0.0017, CA1.2 vs CA1.4 *P*_*adjust*_ = 0.002; **l**: CA1.1 vs CA1.4 *P*_*adjust*_= 0.0007, CA1.2 vs CA1.4 *P*_*adjust*_ = 0.0005, CA1.3 vs CA1.4 *P*_*adjust*_ = 0.0003. *P < 0.05, **P < 0.01, ***P < 0.001, ****P < 0.0001.

A unique aspect of single-cell data is the ability to evaluate cell states. We explored translational cell states across CA3 neurons, which had the most genes clustered by cell type (Extended Data Fig. 5c). CA3 did not have subclusters defined by RNA (Fig. 3b). Therefore, we evaluated the distribution of total EPR per cell. We observed a bimodal distribution for each replicate (Fig. 4a), suggesting ‘high’ and ‘low’ translational states. We clustered neurons on the basis of the combined distribution across replicates into high and low translational states (Fig. 4b). High translation neurons had significantly higher total EPR and number of edited genes, as expected (Fig. 4c,d), and this observation remained true when three replicates were analysed separately (Extended Data Fig. 6a,b). Total RNA counts per cluster were not significantly changed, indicating that the EPR differences within CA3 cells are not driven by RNA expression (Fig. 4e and Extended Data Fig. 6c). Ribo-STAMP RNA levels were also higher in high translation neurons (Fig. 4f and Extended Data Fig. 6d). However, filtering for CA3 neurons in the high and low translation clusters with similar (greater than 0.65 and less than 0.8 quantiles) Ribo-STAMP RNA levels still resulted in higher EPR levels for the high translation cluster (Extended Data Fig. 6e), suggesting that Ribo-STAMP levels are not driving the high translational state. Gene Ontology enrichment analysis of genes with significant EPR enrichment in the high translation cluster revealed biological processes associated with synaptic function (Fig. 4g and Supplementary Table 5). Consistently, synaptic genes such as *Grin2b*, *Celf4* and *Camk2a* showed increased EPR expression in the high versus low translation cluster, with no difference at the RNA level (Fig. 4h and Extended Data Fig. 6f). Instead, genes that were not significantly changing between high and low translational states were enriched in ‘dendrite development’ and ‘exocytosis’ (Fig. 4g). An example is the splicing factor *Luc7l2*, which showed no difference in either RNA or EPR between high or low translation clusters (Extended Data Fig. 6g). This suggests that high translating CA3 neurons might be characterized by enhanced synaptic activity, highlighting a potential neuronal state.

**Extended Data Fig. 7:**
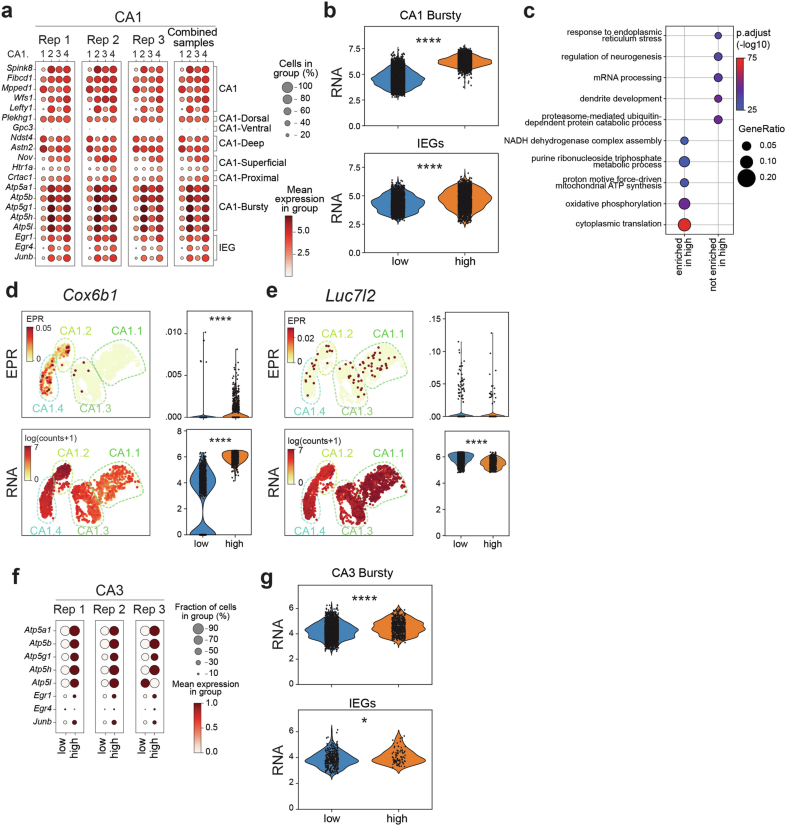
Example genes enriched in translational states in CA3 and CA1 neurons. **a**, Expression of CA1 subfield marker genes, CA1 bursty genes and IEGs in CA1 neurons RNA-defined clusters in individual or combined replicates. **b**, Quantification of CA1 bursty genes and IEGs in CA1 neurons ‘high’ and ‘low’ translation clusters. Shown are normalized RNA counts (log(counts+1) per gene in each gene list per cell. *n* = 760 high cells and 1580 low cells, *n* = 3 mice. *n* = 5 bursty genes and 3 IEGs. **c**, GO analysis of EPR-enriched genes in the CA1 ‘high’ translation cluster versus non-enriched genes. Enriched in high, *n* = 702; Not enriched in high, *n* = 1610. Hypergeometric test, Benjamini–Hochberg correction. **d**,**e**, Expression of example genes from GO terms analysis in (**c**), enriched in CA1 ‘high’ translation cluster *Cox6b1* (**d**), or not enriched, *Luc7l2* (**e**), shown as EPR and RNA (log(counts+1)) UMAP (left) and quantifications (right). **f**, Expression of CA1 bursty genes and IEGs in CA3 neurons ‘high’ and ‘low’ translation clusters in individual replicates. **g**, Quantification of CA1 bursty signature and IEGs in CA3 neurons ‘high’ and ‘low’ translation clusters. (**a**,**f**) Dotplot colors represent mean log(counts+1), size of bubble represent % of cells in group. (**d**,**e**) UMAPs: UMAP1 vs UMAP2. (**b**,**g**) Two-sided Mann-Whitney U test; **b**: CA1 Bursty *P* < 1 × 10^−308^; IEG *P* = 6.05 × 10^−42^, **g**: CA3 Bursty *P* = 2.65 × 10^−25^; IEGs *P* = 2.61 × 10^−3^. (**d**,**e**) Two-sided Mann-Whitney U test, Benjamini–Hochberg correction, **d**: Cox6b1 EPR *P*_*adjust*_ = 8.51 × 10^−40^; Cox6b1 RNA *P*_*adjust*_ < 1 × 10^−308^; **e**: Luc7l2 RNA *P*_*adjust*_= 2.53 × 10^−111^. *P < 0.05, ****P < 0.0001.

Next, we explored whether RNA-defined clusters in a different cell type reflected translational states, focusing on the four CA1 subclusters (Extended Data Fig. 6h). We found that CA1.2 displayed increased total EPR, whereas CA1.2 and CA1.4 showed increased number of edited genes (Extended Data Fig. 6i–l). Thus, we defined CA1.2 and CA1.4 as high CA1 translation clusters and CA1.1 and CA1.3 as low CA1 translation clusters. We observed that the number of edited genes and total EPR were significantly higher in the high translation cluster (Extended Data Fig. 6m–o). To understand what may be driving the difference in translational rate between CA1 high and low clusters, we looked at CA1 subfield marker genes and well-characterized IEGs. We found that the high translation clusters were enriched in ‘bursty’, a distinct neuronal firing pattern characterized by rapid bursts of action potentials, as well as marker genes such as *Atp5a1* and IEG such as *Egr1* (Extended Data Fig. 7a,b). Gene Ontology analysis revealed that the top significantly edited genes in the high translation cluster were involved in mitochondrial function (Extended Data Fig. 7c and Supplementary Table 5). Among these, mitochondrial gene *Cox6b1* was preferentially edited in the high translation clusters (Extended Data Fig. 7d). *Luc7l2* showed no differences (Extended Data Fig. 7e). Bursty firing in the hippocampus is known to be critical for learning and memory^41^, and mitochondria generate the cellular energy needed to support synaptic transmission. Unbiased translational mapping with Ribo-STAMP supports these findings. We found that CA3 high translation neurons also exhibited higher bursty and IEG RNA expression (Extended Data Fig. 7f,g). Altogether, this suggests that CA3 and CA1 high translation neurons may be undergoing increased activity, including potentially bursty firing patterns.

## Transcript and isoform translation in single cells

**Extended Data Fig. 8:**
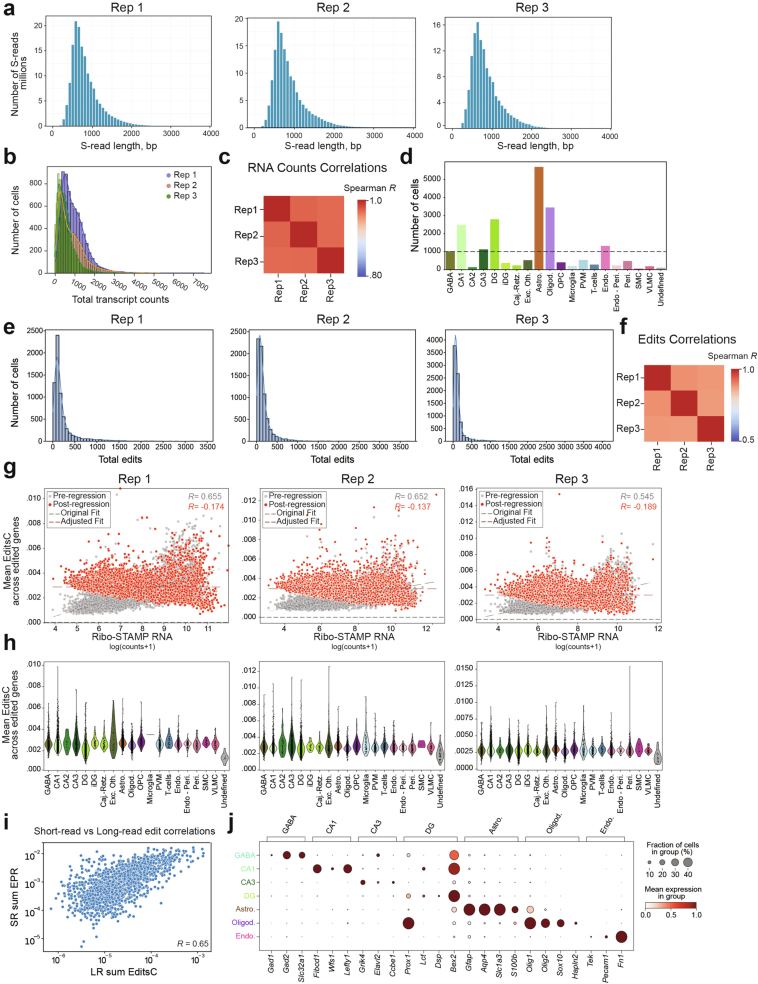
Expression and translation quality controls for long-read RNA-seq in the mouse hippocampus. **a**, Distribution of segmented reads (S-reads) by S-read length for the three replicates. **b**, Distribution of cells by transcript counts for the three replicates. **c**, Transcript RNA counts correlations across replicates (Spearman correlation; Rep1 vs Rep2, *R* = 0.97; Rep1 vs Rep3, *R* = 0.97; Rep2 vs Rep3, *R* = 0.97). **d**, Number of cells retained per cell type. **e**, Distribution of cells by Edits for the three replicates. **f**, Edits correlations across replicates (Spearman correlation; Rep1 vs Rep2, *R* = 0.89; Rep1 vs Rep3, *R* = 0.88; Rep2 vs Rep3, *R* = 0.87). **g**, Scatter plot of EPR by Ribo-STAMP RNA expression log(counts+1) before and after performing regression. Red, original EPR values; Gray, post-regression normalized EPR values. **h**, Mean EditsC per cell across edited genes for each replicate. **i**, Correlation between short-read EPR and long-read EditsC (Spearman correlation, *P* < 10^−5^, *R* = 0.65). **j**, Cell type-specific expression of known marker genes in the long-read dataset. Colors represent mean log(counts+1) standardized by row, size of bubble represent % of cells in group. (**d**,**h**,**j**) GABA, GABAergic neurons; DG, dentate gyrus; iDG, DG immature; Caj.-Retz., Cajal Retzius; Exc. Other, excitatory neurons unassigned; Astro., astrocytes; Oligod., oligodendrocytes; OPC, oligodendrocyte precursor cells; Micro., microglia; PVM, perivascular macrophages; Imm. Other, Immunitary cells, other; Endo., endothelial cells; Endo.-Peri., endothelial cells- pericytes; Peri., pericytes; SMC, smooth muscle cells; VLMC vascular leptomeningeal cells.

Neurons exhibit high levels of alternative splicing to generate a rich repertoire of RNA isoforms and proteforms^5,9^. To understand the mechanistic roles of these transcripts, it is necessary to map isoforms that are preferentially translated or undergoing translational control. However, so far, technological barriers limit studying transcript-specific and isoform-specific translation in single cells. Thus, we coupled Ribo-STAMP with long-read scRNA-seq to capture translational regulation of specific transcripts and isoforms at single-cell resolution. We leveraged MAS-ISO-seq^35^ to perform long-read sequencing on PacBio’s Revio platform. RNA counts from each sample were highly correlated to each other, and the average length of each segmented read was 0.843 kb (Extended Data Fig. 8a–c), slightly shorter than a previous regular ISO-seq dataset (0.998 kb)^14^. Cells profiled by MAS-ISO-seq were assigned to cell types on the basis of short-read RNA-seq data from the same cells (Extended Data Fig. 8d). To analyse transcript-specific translation, we used MARINE to identify isoform-specific and cell-type-specific C-to-U edits in the long-read data. We calculated the ratio of edited Cs to all Cs contained within exons of an RNA transcript, in addition to normalizing by reads, which we term EditsC. Before calculating EditsC, we removed all edits that overlapped with annotated SNPs. Edits from each sample were highly correlated to each other (Extended Data Fig. 8e,f). We found that EditsC was correlated to Ribo-STAMP expression. Thus, we performed linear regression for Ribo-STAMP similar to the short-read data (Extended Data Fig. 8g). Mean EditsC across cell types was similar to the short-read dataset (Extended Data Fig. 8h), and we found a significant correlation between the long-read EditsC dataset and the short-read EPR dataset (Extended Data Fig. 8i), indicating that the two sequencing methods are comparable and detect mostly the same edits. For downstream analysis, we filtered cells by a minimum RNA count and minimum edit threshold. We analysed only cell types with more than 1,000 cells: GABA, CA1, CA3 and dentate gyrus neurons, astrocytes, oligodendrocytes and endothelial cells (Extended Data Fig. 8d,j). The remaining cell types expressed the marker genes akin to those obtained from short-read sequencing (Extended Data Fig. 8j).

**Fig. 5:**
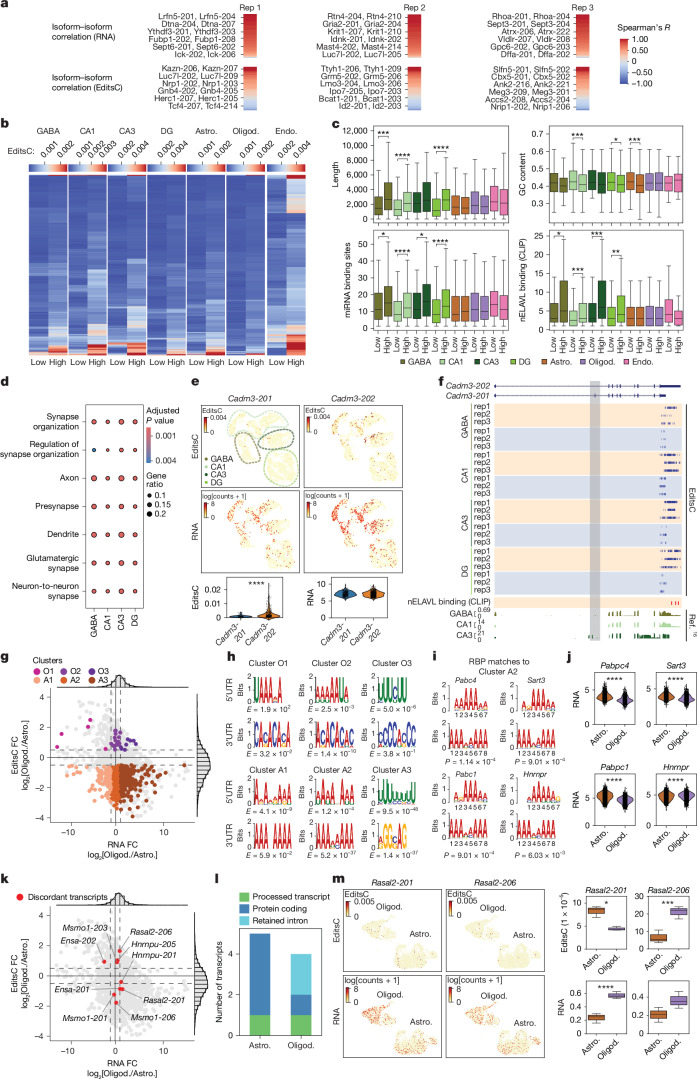
**Ribo-STAMP detects transcript-specific and isoform-specific translation in single cells.** **a**, RNA counts and EditsC pairwise Spearman correlations between isoforms from the same gene across cell types. **b**, EditsC for isoforms from the same gene with highest and lowest translation. Mean EditsC, *n* = 3 mice. **c**, Transcript feature analyses of 3′ UTRs from transcripts in **b**. *n* = number of transcripts per cell type: GABA, 182; CA1, 768; CA3, 183; DG, 478; Astro., 767; Oligod., 619; Endo., 94. *P* values in Supplementary Table 6. **d**, Gene Ontology analysis of transcripts in **b** for neuronal cell types. All ontologies were selected. Hypergeometric test; Benjamini–Hochberg correction. **e**, EditsC and RNA UMAPs (top) and quantifications (bottom) for isoforms *Cadm3-201* and *Cadm3-202*. EPR *P* = 2.19 × 10^−7^. **f**, IGV browser tracks showing edits detected on *Cadm3-201* (blue) and *Cadm3-202* (orange) isoforms. nELAVL CLIP binding sites highlighted in red. Tracks from RiboTag (bottom). *Cadm3-202*-specific exon is highlighted in grey. **g**, Differential EditsC and RNA expression analysis for Oligod. versus Astro. Significant EditsC enrichment: log_2_FC > 0.5 or < −0.5; Welch’s *t*-test; *P* < 0.05. Significant RNA enrichment: two-sided Mann–Whitney *U*-test; Benjamini–Hochberg correction; *P*_adjust_ < 0.01. Oligod. (O clusters; EditsC log_2_FC > 0.5): RNA log_2_FC: O1 < 1, O2 between ±1, O3 > 1. Astro. (A clusters; EditsC log_2_FC < −0.5): RNA log_2_FC: A1 < 1; A2 between ±1, A3 > 1. **h**, Top enriched motifs found in each cluster. **i**, Example RBPs enriched motifs on 3′ UTR top enriched motif in cluster A2. **j**, RNA expression of RBPs found in **i** in Oligod. and Astro. *Pabpc4*, *P* = 1.39 × 10^−111^; *Pabpc1*, *P* = 3.238 × 10^−21^; *Sart3*, *P* = 1.02 × 10^−61^; *Hnrnpr*, *P* = 7.81 × 10^−10^. **k**, Same scatterplot as in **g**, highlighting isoforms of the same gene undergoing significant differential translation in Oligod. versus Astro.; two-sample, unequal variance *t*-test; *P* < 0.05. **l**, Transcript biotypes of isoforms in **k** regardless of *P*. **m**, EditsC and RNA UMAPs (left) and quantifications (right) for isoforms *Rasal2-201* and *Rasal2-206*. EditsC: Welch’s *t*-test, mean EditsC per animal, *n* = 3 mice, *Rasal2-201 P* = 0.02 and *Rasal2-206 P* = 8.40 × 10^−3^. RNA: two-sided Mann–Whitney *U*-test; Benjamini–Hochberg correction; *n* = 5,695 Astro. cells and 3,450 Oligod. cells; *n* = 3 mice; *Rasal2-201 P*_adjust_ = 3.45 × 10^−4^. Box plots show the interquartile range, with the line inside marking the median (**c**,**m**). UMAPs: UMAP1 versus UMAP2 (**e**,**m**). Two-sided Mann–Whitney *U*-test; *P < 0.05; **P < 0.01; ***P < 0.001; ****P < 0.0001 (**c**,**e**,**j**).

**Extended Data Fig. 9:**
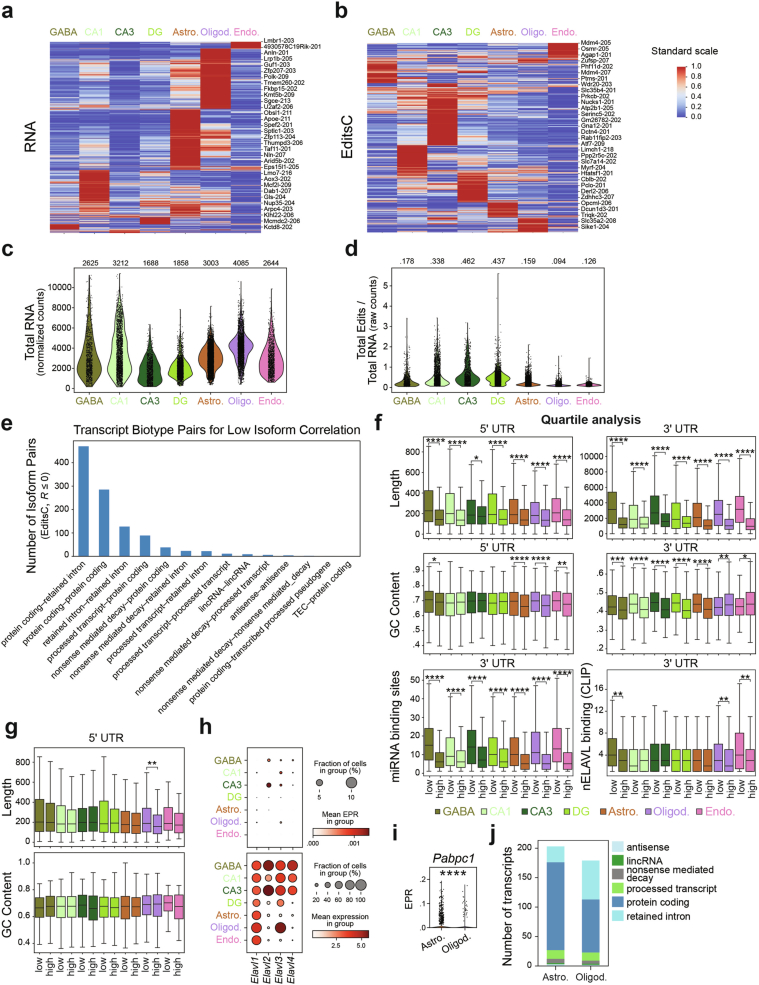
Cell type- and transcript-specific expression and editing analysis. **a**,**b**, Heatmap of RNA expression (log(counts+1), *n* = 15,967 transcripts) (**a**) and transcript edits (EditsC, *n* = 3,857 transcripts) (**b**). Shown is EditsC and RNA counts standardized by row. **c**, Total RNA counts per cell type. **d**, Total edits divided by total raw RNA counts per cell type. **e**, Transcript biotypes of isoform pairs with EditsC correlation ≤ 0 from (Fig. 5a). **f**, Gene features analyses of ‘high’ and ‘low’ translation quartiles for each cell type comparison. miRNAs and nELAVL binding sites in 3′UTRs. **g**, 5′UTR gene features analyses from ‘high’ and ‘low’ isoform from the same gene as in (Fig. 5c). **h**, EPR (top) and RNA expression (bottom) of the *Elavl* transcripts. **i**, EPR levels of *Pabpc1* in oligodendrocytes and astrocytes. **j**, Transcript biotypes of isoforms from (Fig. 5k) enriched in oligodendrocytes or astrocytes regardless of *p*-value. (**f**,**g**) Boxplots show the interquartile range, with the line inside marking the median. Whiskers extend up to 1.5 times the interquartile range from the lower and upper quartiles. Statistics reflect significant differences between high and low transcripts in each cell type; Mann-Whitney U test; *n* for (**f**) = number of transcripts per cell type; GABA = 618, CA1 = 1511, CA3 = 686, DG = 1212, Astro.=1611, Oligod.=1243, Endo.=402; *n* for (**g**) = number of transcripts per cell type; GABA = 182, CA1 = 768, CA3 = 183, DG = 478, Astro. = 767, Oligod. = 619, Endo. =9 4. (**f**,**g**,**i**) Two-sided Mann-Whitney U test, **f** and **g**: p-values in Supplementary Table 6, **i**: *P* = 3.23×10^−21^. *P < 0.05, **P < 0.01, ***P < 0.001, ****P < 0.0001.

To identify cell-type-specific transcript expression and translation, we performed pseudobulk clustering of transcripts by both RNA counts and EditsC (Extended Data Fig. 9a–d). We considered only transcripts from genes with multiple RNA isoforms represented in the data. We collected counts for all expressed transcripts. When correlating isoform RNA counts and EditsC from the same gene across cell types, we found that most of the RNA counts for isoform pairs (two isoforms from the same gene) were positively correlated, whereas EditsC showed reduced correlation (Fig. 5a). Indeed, 37% of isoform pairs had an EditsC correlation of 0 or below compared with 1.3% for RNA counts. Almost half of these 37% isoform pairs were ‘protein coding to retained intron’ isoforms (Extended Data Fig. 9e). Intron retention is known to increase protein diversity in the brain through regulation of mRNA stability and subcellular localization in response to neuronal activity^42^. Our findings suggest that these overlooked transcripts may contribute to cell-type-specific gene expression regulation.

To evaluate how alternative splicing affects translation, we first compared high and low translation of all transcripts using EditsC for each cell type. We characterized transcript length, 5′ and 3′ UTR GC content and 3′ UTR miRNA binding sites, features well established to regulate translation^43,44^. We observed that the high translation quartile for most cell types had decreased 5′ and 3′ UTRs, GC content and miRNA binding sites (Extended Data Fig. 9f and Supplementary Table 6), recapitulating previous findings^39^ indicating that longer UTR regions harbour more *cis*-regulatory elements affecting mRNA translation^34,45^. Moreover, high GC content in 5′ and 3′ UTR can result in complex RNA structures that cause inefficient ribosome or RBP scanning, leading to decreased RNA translation and expression^43^. Next, we asked if the same regulatory rules applied to isoforms from the same gene. For each cell type, we identified the isoforms from the same gene that had the ‘highest’ and ‘lowest’ EditsC (Fig. 5b). CA1, astrocytes and oligodendrocytes displayed the highest number of isoform pairs, most probably because these are the most represented cell types in the dataset. In addition, 5′ and 3′ UTR length, GC content and miRNA binding sites were mostly unchanged in non-neuronal cells (Fig. 5c, Extended Data Fig. 9g and Supplementary Table 6). We found that all neuronal subclasses, except CA3, displayed significantly increased 3′ UTR length and miRNA binding sites, with CA1 and dentate gyrus neurons also showing decreased GC content in the highest edited isoforms (Fig. 5c). These findings suggest that isoforms with longer 3′ UTRs in neurons potentially contain binding motifs for positive translational regulators. Neuron-specific ELAV-like proteins (nELAVLs) interact with initiation factors to promote translation^46^. We confirmed that nELAVLs are selectively expressed in neuronal cells, as opposed to their ubiquitously expressed paralogue ELAVL1 (Extended Data Fig. 9h). Notably, nELAVL binding sites were increased in the highest edited isoforms selectively in neuronal cells (Fig. 5c), a pattern that was not reflected in the high versus low translation quartile analysis (Extended Data Fig. 9f). Gene Ontology enrichment analysis showed that the neuronal genes from the highest versus lowest analysis are primarily involved in synaptic biology (Fig. 5d), in line with the well-established link between isoform complexity and synapse biology^47^. Of the transcripts shared across neurons, we highlighted two isoforms of *Cadm3*, a cell adhesion molecule important for synapse formation^48^ (Fig. 5e). Across neurons, *Cadm3-202* contains a longer 3′ UTR, displays higher edits and contains unique nELAVL binding sites compared with *Cadm3-201* (Fig. 5f). A cell-type-specific RiboTag dataset^16^ further showed little coverage across an exon present in *Cadm3-201* but not in *Cadm3-202* (Fig. 5f, bottom), suggesting lower *Cadm3-201* translation and thus validating our finding, together with other examples (Supplementary Table 7).

To characterize differential transcript translation in different cell types, we performed pairwise comparisons of EditsC between all cell-type pairs. Owing to high cell numbers, the oligodendrocytes versus astrocytes comparison displayed the most differentially translated transcripts, with astrocyte transcripts exhibiting the most significant editing (Fig. 5g and Supplementary Table 8). We evaluated transcript EditsC and RNA counts and defined six groups of differential translation and expression (Fig. 5g and Supplementary Table 9). To identify putative translational regulatory elements within these groups, we performed motif enrichment on the 5′ and 3′ UTR regions. We observed significant motifs for each group (Fig. 5h), with A-rich motifs in both 5′ and 3′ UTRs for astrocytic clusters, which resemble the binding sites for RBPs with known A-rich motif binding and splicing functions^49–51^ (Fig. 5i). Of these, *Pabpc4*, *Sart3* and *Pabpc1* were significantly enriched in astrocytes, whereas *Hnrpnr* was enriched in oligodendrocytes (Fig. 5j). EPR for *Pabpc1* was also enriched in astrocytes (Extended Data Fig. 9i). These RBPs facilitate translation^50^, suggesting possible mechanisms underlying the increased translation in astrocytes.

We next sought to identify alternative isoforms of the same gene undergoing differential translation in different cell types. We observed 17 transcripts from eight genes that displayed significantly discordant isoform translation in one cell type compared with another (Supplementary Table 9). Oligodendrocyte versus astrocyte had the most significantly discordant isoforms, with protein-coding isoforms harbouring more editing in astrocytes, whereas isoforms retaining introns in the same gene were more edited in oligodendrocytes (Fig. 5k,l and Extended Data Fig. 9j). For example, *Rasal2-201*, a protein-coding isoform, had increased EditsC in astrocytes, but *Rasal2-202*, a retained intron isoform, had increased EditsC in oligodendrocytes (Fig. 5m). RNA counts for *Rasal2-201* were significantly higher in oligodendrocytes, whereas RNA counts for *Rasal2-202* were not significantly different. This suggests that oligodendrocytes may selectively use retained introns as a cell-type-specific mechanism of translational regulation.

## Differential translation in CA1 and CA3

**Fig. 6:**
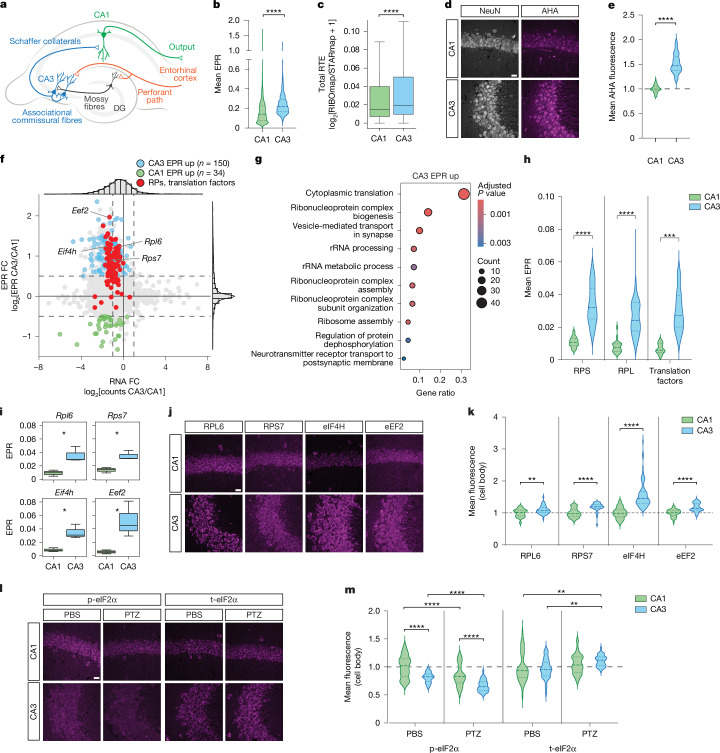
**Differential translation between CA1 and CA3 neurons.** **a**, Schematic of the hippocampal circuit. **b**, Mean EPR values across CA1 and CA3 neurons. **c**, Total RTE in CA1 and CA3 neurons from RIBOmap. Lines represent the median, and whiskers indicate quartiles. **d**, Representative images of AHA-labelled CA1 and CA3 neurons. **e**, Mean fluorescence intensity quantification of AHA signal from **d**, normalized to CA1; *n* = 90 cells per condition; *n* = 3 mice. **f**, Differential EPR and RNA analysis of CA3 versus CA1. Significant EPR enrichment: bootstrap test (Methods), *P* < 0.05; log_2_FC > 0.5 or < −0.5. RPs, translation factors in red. **g**, Gene Ontology analysis from CA3 EPR upregulated genes in **f**. Hypergeometric test; Benjamini–Hochberg correction. **h**, Mean EPR of CA3 EPR upregulated RPs and translation factors. **i**, Mean EPR of CA3 EPR upregulated candidates. **j**, Representative IHC images of CA3 EPR upregulated candidates in CA1 and CA3 areas. **k**, Mean fluorescence intensity quantification normalized to CA1; *n* = 45 cells (RPL6, RPS7 and eEF2) and *n* = 72 cells (eIF4H) from *n* = 3 mice. **l**, Representative IHC images of p-eIF2α and t-eIF2α in CA1 and CA3 with PBS or PTZ. **m**, Mean fluorescence intensity quantification normalized to PBS-treated CA1; *n* = 45 cells per condition from *n* = 3 mice. Box plots show the interquartile range, with the line representing the median (**c**,**i**). Whiskers extend up to 1.5 times the interquartile range from the lower and upper quartiles. Two-sided Mann–Whitney *U*-test (**b**,**c**,**e**,**k**). *P* < 0.0001 (**b–e**). *P* < 0.0001, except RPL6 (*P* = 0.004) (**k**). RPS and translation factors, two-sided *t*-test and RPL and two-sided Mann–Whitney *U*-test; *P* < 0.0001, except translation factors (*P* = 0.0142) (**h**). Bootstrap test; *Rpl6*, *P* = 0.0264; *Rps7*, *P* = 0.0208; *Eif4h*, *P* = 0.0262; *Eef2*, *P* = 0.0427 (**i**). Two-way ANOVA followed by Tukey’s multiple comparison post hoc test; *P* < 0.0001, except t-eIF2a CA1 PBS versus PTZ *P* = 0.0081, CA3 PBS versus PTZ *P* = 0.0024 (**m**). *P < 0.05; **P < 0.01; ***P < 0.001; ****P < 0.0001. Violin plots show median with top and bottom quartiles. Scale bars, 20 μm.

CA1 and CA3, along with dentate gyrus, are the main excitatory neurons in the hippocampal trisynaptic circuit (Fig. 6a). These neurons share similar morphology; however, they differ significantly in their activity dynamics and contributions to learning and memory. Previous studies have scarcely explored genome-wide translation or protein differences between CA1 and CA3 (refs. ^52,53^), with none noting the translational upregulation in CA3 that we observed using short-read and long-read Ribo-STAMP analysis (Extended Data Figs. 5c and 9b). Our findings prompted further investigation into these distinct cell types. To compare CA1 with CA3, we grouped all four CA1 subtypes into one group called CA1. First, we measured EPR per gene and found that CA3 displays increased translation compared with CA1 (Fig. 6b). We validated this finding by calculating the relative translational efficiency (RTE) for CA1 and CA3 using the RIBOmap and STARmap datasets^27^ and found that total RTE levels were higher in CA3 than in CA1 (Fig. 6c). Further, we measured newly synthesized proteins in hippocampal slices using fluorescent non-canonical amino acid tagging (FUNCAT) and found that CA3 showed higher basal incorporation of the methionine analogue l-azidohomoalanine (AHA; Fig. 6d,e).

**Extended Data Fig. 10:**
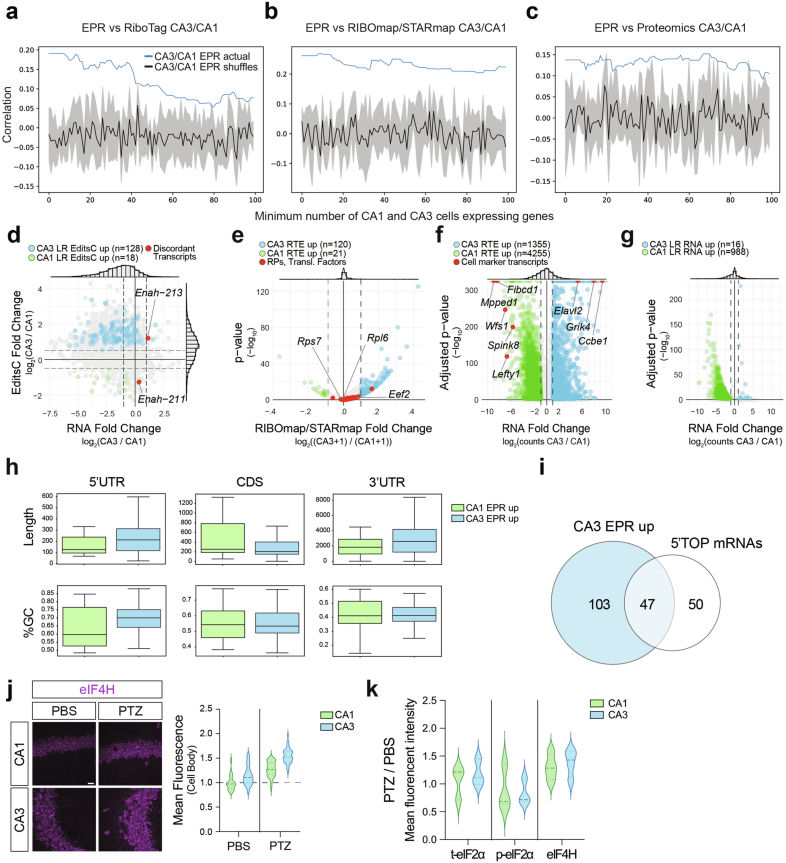
Validations of CA1 and CA3 EPR and RNA changes. **a-c**, Spearman correlation between CA3/CA1 EPR log_2_FC and RiboTag, RIBOmap/STARmap or proteomics datasets (blue line). The gray area represents the range of mean ± 1 standard deviation derived from shuffled datasets (*n *= 100 shuffles). The black line indicates the mean correlation across shuffled datasets, serving as a null distribution. **d**, Long-read EditsC and RNA differential expression analysis comparing CA3 vs CA1 (Significant EditsC enrichment: two-sample, unequal variance *t*-test *P* < 0.05, log_2_FC > 0.5 or <−0.5; Significant RNA enrichment: Two-sided Mann-Whitney U test, Benjamini–Hochberg correction, *P*_*adjust*_ < 0.01, log_2_FC > 1 or <−1). *Enah* is the only gene with significantly discordant isoforms between the two cell types. **e**, RIBOmap/STARmap differential expression analysis comparing CA3 vs CA1 (Significant enrichment: Two-sided Mann-Whitney U test, *P* < 0.05, log_2_FC > 0.1 or <−0.1). Ribosomal proteins, translation factors and Ribo-STAMP candidates validated in Fig. 6j,k are highlighted. **f**, Short-read differential RNA expression analysis comparing CA3 vs CA1 expressed genes (Significant RNA enrichment: Two-sided Mann-Whitney U test, Benjamini–Hochberg correction, *P*_*adjust*_ < 0.01, log_2_FC > 1 or <−1). Cell type-specific known gene markers are highlighted. **g**, Long-read differential RNA expression analysis comparing CA3 vs CA1 (Significant RNA enrichment: Two-sided Mann-Whitney U test, Benjamini–Hochberg correction, *P*_*adjust*_ < 0.01, log_2_FC > 1 or <−1). **h**, Transcript feature analysis for CA3 and CA1 EditsC-enriched transcripts. Boxplots show the interquartile range, with the line inside marking the median. Whiskers extend up to 1.5 times the interquartile range from the lower and upper quartiles. Two-sided Mann-Whitney U test; *n* = 18 CA1 transcripts and *n* = 128 CA3 transcripts. **i**, Overlap between CA3 EPR upregulated and 5′TOP motifs containing mRNAs. **j**, Left, representative IHC images of eIF4H in CA1 and CA3 areas with PBS or PTZ. Right, mean fluorescence intensity quantification normalized to PBS-treated CA1. *n* = 60 cells per condition were analyzed from *n* = 2 mice. **k**, Mean fluorescence intensity quantification of p-eIF2α, t-eIF2α and eIF4H levels in CA1 and CA3 excitatory neurons from Fig. 6m and Extended Data Fig. 10j, shown as PTZ / PBS ratio. Violin plots show median with top and bottom quartiles.

To understand which genes were differentially translated between CA1 and CA3, we performed differential EPR analysis (Fig. 6f). We then compared CA3/CA1 EPR log_2_FC with RiboTag^18^, RIBOmap/STARmap^30^ and human proteomics datasets (Extended Data Fig. 10a–c) and found that our data positively correlated with all three existing datasets. To identify translational differences, we focused on genes showing significant CA3/CA1 EPR change, revealing an enrichment of differentially translated genes in CA3 versus CA1 (Fig. 6f and Supplementary Table 10). Notably, 37% of genes showing EPR changes featured modest RNA changes and would thus remain undetected with scRNA-seq. Overall, this evidence aligns with our data showing global translational increase in CA3 (Fig. 6b) and was corroborated at the transcript level with long-read sequencing (Extended Data Fig. 10d) and by the RIBOmap/STARmap dataset (Extended Data Fig. 10e and Supplementary Table 10). The number of DEGs identified by short-read sequencing and transcripts detected by long-read sequencing was higher in CA1 than in CA3 (Extended Data Fig. 10f,g and Supplementary Table 10).

To understand the biological function of CA3 differential translation, we performed Gene Ontology term analysis. The most enriched term was related to the translational machinery (Fig. 6g and Supplementary Table 11). We observed higher mean EPR for ribosomal proteins and translation factors in CA3 versus CA1 neurons (Fig. 6h and red data points in Fig. 6f). To validate these findings, we measured EPR and endogenous protein expression of selected ribosomal proteins and translation factors and confirmed a significant enrichment in CA3 versus CA1 neurons (Fig. 6i–k). We did not observe enrichment for the same genes in the RIBOmap/STARmap dataset (Extended Data Fig. 10e), suggesting that Ribo-STAMP may confer a higher level of sensitivity. Looking at the transcript features of genes with higher EPR in CA1 and CA3, we did not observe significant differences in 5′ UTR, CDS and 3′ UTR length or GC content (Extended Data Fig. 10h). Notably, 31% of the genes with higher EPR in CA3 contained 5′ terminal oligopyrimidine (5′TOP) motifs (this was not the case for genes with higher EPR in CA1), which are commonly found in translational machinery genes (Extended Data Fig. 10i and Supplementary Table 12). Altogether, these data suggest that CA3 exhibits higher global translational rates, which may result in higher protein levels compared with CA1.

To explore if differential translation between CA1 and CA3 may be influenced by neuronal activity, we measured phosphorylation levels of the α-subunit of eIF2 (p-eIF2α), which decrease upon activity-induced translation^54^. We found that p-eIF2α basal levels were lower in CA3 compared with CA1 neurons, whereas the total eIF2α (t-eIF2α) levels were unchanged (Fig. 6l,m). This result validates our EPR and FUNCAT data (Fig. 6b,d,e), showing that CA3 exhibits higher protein synthesis than CA1 and suggesting a potential contribution of neuronal activity. To test this, we induced a robust increase in neuronal activity using pentylenetetrazole (PTZ), a GABA receptor antagonist, known to elicit epileptic seizures. Compared with phosphate-buffered saline (PBS)-treated mice, PTZ-treated mice showed downregulation of p-eIF2α levels, with similar relative changes in CA3 and CA1 neurons (Fig. 6l,m and Extended Data Fig. 10j). Under the same conditions, t-eIF2α levels remained unchanged, except in CA3 neurons after PTZ treatment in which they were increased. Further, the translation factor eIF4H was induced in both CA1 and CA3 after PTZ treatment, with overall protein expression higher in CA3 (Extended Data Fig. 10j,k). Our data suggest that both CA1 and CA3 have the capacity to induce translation after strong neuronal stimulation. However, at baseline, CA3 undergoes higher translation, which may be attributable to decreased p-eIF2α.

## Discussion

Ribo-STAMP enabled the characterization of neuronal activity (Fig. 2) and cell-type-specific translation (Fig. 3) in the brain. We discovered that CA1 and CA3 neurons have high and low relative translational states (Fig. 4). On the basis of the enrichment of metabolism and synaptic genes in the high translation neurons, we speculate that they are potentially responding to environmental factors (such as neuronal activity), which increase translation of these pathways^55^. Accordingly, previous studies have found that CA3 contains 17% of cells with bursty firing patterns^56^. We found that 18% of CA3 cells belong to the high translation cluster, suggesting that Ribo-STAMP efficiently detects cell states within a neuronal subtype.

This study demonstrates, to our knowledge, the first analysis of transcript-specific and isoform-specific translation in single cells, a gene expression feature that is normally omitted by scRNA-seq analysis. Long-read sequencing enabled the identification of isoforms undergoing differential translation within and across cell types (Fig. 5). Comparing translation of isoforms from the same gene, we found that highly translated isoforms in neurons have longer 3′ UTRs and encode synaptic proteins, consistent with previous evidence^2^. These longer isoforms contain more regulatory elements than their shorter counterparts. Increased translation may be attributable to an enrichment of binding sites for nELAVLs, which are positive regulators of RNA stability and translation^46^.

Similarly, differential transcript translation analysis between astrocytes and oligodendrocytes revealed higher translation for astrocyte transcripts, which were enriched for *Pabpc1* binding motifs, an RBP that stabilizes translation. We also detected divergent translation of alternative isoforms from the same genes in astrocytes and oligodendrocytes. Higher translated isoforms in oligodendrocytes contain retained introns, a feature with repressive effects on translation and of monosome-associated rather than polysome-associated transcripts. This suggests possible cell-type-specific translation of intron-retaining isoforms, a hypothesis requiring extensive future experiments to be validated.

Finally, we identified differences in translational efficiency between CA1 and CA3 that would have been missed by scRNA-seq. Despite CA1 having more DEGs and isoforms (Extended Data Fig. 10), CA3 had increased global translation (Fig. 6). We speculate that the increased translation in CA3 could be attributable to an increased response to neuronal activity^54^ or higher metabolism and nutrient availability at basal state^57^. These deeply interlinked pathways impinge on the translation of 5′TOP motifs^58,59^, contained in ribosomal proteins and translation factors, to regulate global protein synthesis. Future studies will elucidate the mechanisms and implications of increased translation in CA3.

The limitations of Ribo-STAMP include reliance on sequencing depth and RNA base editing efficiency for translational measurements. Future efforts will focus on improving long-read sequencing and identifying enzymes with increased editing specificity and rate^32^. Our easily adoptable tool will enable the systematic characterization of cell-type-specific and isoform-specific translation at multiple scales, from subcellular proteomes (synapses from different cell types^60^) to cellular function, behaviour or disease.

## Methods

### Molecular cloning and AAV production

The original Ribo-STAMP construct consisted of a Tet-On hRPS2–APOBEC lentiviral expression vector, with a human phosphoglycerate kinase (PGK) promoter driving the expression of rtTA and pac, designed for generation of stable human cell lines^18^ (Fig. 1b). ORFs of Ribo-STAMP variants were first cloned into a plasmid derived from the pLIX_403 vector (a gift from D. Root; Addgene plasmid no. 41395) containing a P2A-mRuby fluorescent protein sequence in-frame with the fusion protein. The RPS2–APOBEC fusion sequence was taken from the pLIX403_Capture1_RPS2_APOBEC_HA_P2A_mRuby construct, as used previously^18^. Following the EGFP-tagging methodology of the TRAP system^61^, the ORF isoform 1 of the mouse Rpl10a was fused to the C terminus of APOBEC1. A similar strategy was adopted for the Rpl22–APOBEC fusion sequence whereby the mouse Rpl22 ORF was fused to the N terminus of APOBEC to mimic the haemagglutinin-tagging strategy of RiboTag^62^. Both fusion sequences were generated as synthetic gBlocks (Integrated DNA Technologies) and cloned into the pLIX403_Capture1_APOBEC_HA_P2A_mRuby parental backbone using Gibson assembly. The Tet-On Ribo-STAMP constructs used in Figs. 1 and 2 were cloned into a mammalian gene expression AAV vector (VectorBuilder) containing a truncated woodchuck hepatitis virus post-transcriptional regulatory element (WPRE) and bovine growth hormone polyadenylation signal (BGH polyA), using Gibson assembly. The tetracycline-responsive promoter element, followed by Ribo-STAMP variant ORFs, was polymerase chain reaction (PCR)-amplified from the previously described plasmids. The hSyn promoter was PCR-amplified from pAAV.Syn.GCaMP6s.WPRE.SV40 (a gift from D. Kim; Addgene plasmid no. 100843), and the rtTA was PCR-amplified from the pLIX_403 vector. Ribo-STAMP constructs used in Figs. 1 and 2 were cloned into a mammalian gene expression AAV vector (VectorBuilder). Constitutive RPS2-STAMP used for in vivo experiments was assembled by VectorBuilder. The construct was cloned into a mammalian gene expression AAV vector (VectorBuilder) containing an EF1A promoter, the RPS2_APOBEC ORF containing an haemagglutinin tag as above and fused with TdTomato through a P2A peptide, followed by a truncated WPRE and BGH polyA. AAVs were prepared following Challis et al.^63^ using the PHP.eB capsid for packaging.

### Animals

C57BL/6J mice were group-housed on a 12-h light–dark cycle and fed a standard rodent chow diet. All experimental protocols were approved by the Institutional Animal Care and Use Committee at Scripps Research Institute and were in accordance with the guidelines from the National Institutes of Health (NIH).

### Primary neuron culture, treatments, infections and viability test

Cortices were dissected from E18 C57BL/6J embryos in Leibovitz’s L-15 Medium (Life Technologies) and incubated in TrypLE Express (Life Technologies) at 37 °C for 4 min. Neurons were mechanically dissociated and plated on poly-d-lysine-hydrobromide-coated six-well plates (600,000 cells per well) or on 12-mm coverslips in 24-well plates (100,000 cells per well). Neurons were maintained in a humidified environment at 37 °C and 5% CO_2_ in neurobasal medium (Life Technologies) supplemented with B27 Plus Supplement (Life Technologies) and GlutaMAX (Life Technologies). At day in vitro (DIV) 3, floxuridine was added to the medium to limit the growth of dividing cells. DIV 6 neurons were infected with 1 × 10^13^ (24-well plates) or 4 × 10^13^ (six-well plates) viral genomes ml^−1^ of inducible Ribo-STAMP AAV virions. Ribo-STAMP expression was induced with 1 µg ml^−1^ Dox for 48 h before collection at DIV 14. BDNF (100 ng ml^−1^) was applied 15 min and 60 min before collection^64,65^. Anisomycin (40 µM) was applied 30 min before collection. For Puro-PLA experiments, neurons were treated with puromycin (10 μm) for 15 min before collection. Cell viability assay was performed using the Cell Counting Kit Assay-8 (MilliporeSigma).

### Proximity ligation assay

Proximity ligation assay (PLA)^65^ was performed using the Duolink kit (Sigma). After puromycin treatment, neurons were washed in PBS, fixed for 10 min in 4% paraformaldehyde (PFA) and washed again in PBS. Fixed neurons were permeabilized with 0.1% Triton X-100 in PBS-CM (PBS (pH 7.4), 1 mM MgCl_2_ and 0.1 mM CaCl_2_) for 10 min. All reactions were incubated in a preheated humidity chamber. After a brief wash with PBS-CM, each sample was blocked with Duolink Blocking Solution for 1 h at 37 °C and then incubated with anti-puromycin and protein-of-interest primary antibodies diluted in Duolink Antibody Diluent overnight at 4 °C. After two washes in wash buffer A, PLA rabbit plus and mouse minus probes were applied at a 1:5 dilution in Duolink Antibody Diluent for 1 h at 37 °C. After washes, ligation and amplification reactions were performed using Duolink Fluorescent Detection Reagent in FarRed. The cells were incubated in the ligation reaction for 30 min at 37 °C, washed with wash buffer A and incubated in the amplification reaction for 100 min at 37 °C. Following the final washes with 1× and 0.01× wash buffer B, cells were postfixed for 10 min with 4% PFA, washed in PBS and prepared for immunocytochemistry.

### Immunocytochemistry

PFA-fixed cells were permeabilized with 0.1% Trixon-X 100 in PBS for 10 min at room temperature, washed in PBS and blocked with 10% normal goat serum (NGS) in PBS for 1 h at room temperature. Cells were incubated with primary antibodies at desired concentrations in 1% NGS in PBS overnight at 4 °C. After three washes with PBS, cells were incubated with secondary antibodies at 1:500 dilution in 1% NGS in PBS for 2 h at room temperature. After three washes with PBS, coverslips were mounted onto microscope slides (Thermo Fisher Scientific) with ProLong Diamond Antifade Mountant (Life Technologies) and imaged.

#### Immunocytochemistry image acquisition and analysis

Representative images in Fig. 1 and Extended Data Fig. 1 were acquired with a Nikon A1 confocal microscope at a 1,024 × 1,024 resolution using a Plan Apo VC 20× DIC N2 objective through the thickness of the neurons (five stacks; 3.2 µM step size). To assess AAV infection, stitched images of four fields were acquired with a Plan Apo 10× DIC L objective, and the percentage of Ribo-STAMP over NeuN-positive neurons was assessed with CellProfiler.

#### Puro-PLA

*Z*-stack images were acquired with a Nikon A1 confocal microscope using a PlanApo ×60 oil, numerical aperture 1.4 objective at a 2,048 × 2,048 resolution throughout the thickness of the neurons (nine stacks; 1.2 µM step size). Five fields within each coverslip were captured, and imaging settings were maintained constant within experiments. Quantification was on the basis of a previous study^65^, with minor modifications. Maximum intensity projections were generated, and manual thresholds were applied to the PLA and MAP2 images using the same values across all samples. In the MAP2-thresholded image, a circular region of interest with a radius of 75 µm, centred on the neuron soma, was selected for quantification, and a mask of the cell body was generated. The mask was dilated by a radius of 2.5 px to include the signal in spines and presynaptic terminals. For each neuron, the area of the dilated MAP2 mask and the area covered by PLA-positive pixels within the mask were quantified. Quantifications are shown as percentage of PLA area over the dilated MAP2 mask area. Statistical tests were performed using GraphPad Prism v.10.2.

### FUNCAT and immunolabelling

The non-canonical amino acid AHA was dissolved in ultra-pure water, and the pH was adjusted to 7.4. PTZ was dissolved in Dulbecco’s phosphate-buffered saline at 5 mg ml^−1^, and the solution was freshly prepared before injections. P25 C57BL/6 male mice were co-injected intraperitoneally with 100 µg gbw^−1^ AHA (added with 10× PBS) and 8.5 µl gbw^−1^ PTZ or 1× PBS. PTZ-treated animals exhibited severe seizures. After 18 h, the mice were anaesthetized with isoflurane and intracardially perfused with PBS, followed by 4% PFA. Brains were postfixed overnight in 4% PFA, washed twice in PBS and cut into 50-μm-thick slices using a vibratome (Leica VT1000S). Click labelling was performed on the basis of the published CATCH protocol, with minor modifications^66^. Slices were incubated in 1 mM CuSO_4_ in ultra-pure water overnight at room temperature with gentle agitation. The slices were transferred in 150 µM CuSO_4_, 300 µM BTTP, 10% dimethyl sulfoxide and 5 μm of AF647 Picolyl Azide in PBS. To kickstart the click reaction, NaAsb was added to a final concentration of 2.5 mM, and the reaction was incubated for 1 h at room temperature with gentle agitation. The reaction was quenched with 4 mM EDTA in PBS (pH 8) and then washed three times in 0.2% Triton X-100 in PBS for 10 min at room temperature. Brain slices were further prepared for IHC.

### Immunohistochemistry

Brain slices (50 μm thick) were blocked in 10% NGS and 0.25% Triton X-100 in PBS for 1 h at room temperature. Slices were incubated in primary antibodies at the desired concentrations in 1% NGS and 0.25% Triton X-100 in PBS overnight at 4 °C on an orbital shaker. After three washes in 0.1% Tween in PBS, the slices were incubated in secondary antibodies at a 1:500 dilution in 1% NGS and 0.25% Triton X-100 in PBS for 2 h at room temperature on an orbital shaker. After three washes in 0.1% Tween in PBS, the slices were mounted on slides with ProLong Diamond Antifade Mountant.

#### IHC acquisition and analysis

Dorsal hippocampus images were acquired in the CA1b and CA3a subfields with a Nikon A1 confocal microscope using a PlanApo ×60 oil, numerical aperture 1.4 objective at a 2,048 × 2,048 resolution. Imaging settings were maintained constant within experiments. For each CA1 and CA3 image, the plane with the brightest signal was selected for quantification. Regions of interest of cell bodies were manually drawn using the NeuN signal as a mask. The mean fluorescence intensity of the analysed proteins within the NeuN mask was measured using Fiji. Interneurons identified through GAD67 staining were excluded from the analysis. Statistical tests were performed using GraphPad Prism v.10.2.

### Ribosome structure analysis

The mouse ribosome structure and the relative location of RPS2, RPL22, RPL10a, transfer RNA (tRNA) and mRNA were accessed from Protein Data Bank (PDB) 7CPU^67^, 6Y0G^68^ and 7LS2 (ref. ^69^). Most of the ribosome structure representation, including ribosomal RNA, tRNA, RPS2, RPL22 and most of the other ribosomal proteins, was generated from PDB 7CPU. Owing to the lack of RPL10a and mRNA on PDB 7CPU, PDB 6Y0G and 7LS2 were superimposed to PDB 7CPU to model the structure and the relative locations of mRNA and RPL10a, respectively. All structure superimposition and figure generation were performed using PyMol (https://www.pymol.org/).

### eCLIP sequencing

eCLIP sequencing was performed following established protocols^70^. Anti-RPS2 antibody (Bethyl A303-794A) was used. Total RNA was taken from samples before starting immunoprecipitation. ribosomal RNA depletion was performed after library preparation (Jumpcode Genomics). Single-end sequencing was performed with 20 million reads per sample on an Illumina NovaSeq at the University of California San Diego (UCSD) Institute for Genomic Medicine. FASTQ files were trimmed for indices using cutadapt 1.18. Trimmed FASTQ files were subsequently aligned to the mm10 genome using the STAR alignment software v.2.5.2b, with the flags --outSAMAttributes All, --outFilterMultimapNmax 10, --outFilterMultimapScoreRange 1, --outFilterScoreMin 10, --alignEndsType EndToEnd. For each gene, eCLIP immunoprecipitation read counts were divided by total read counts to derive an eCLIP-based translational efficiency value. Genes were split into 100 EPR quantiles, and for each quantile’s set of genes, a mean EPR and mean eCLIP-derived translational efficiency value were plotted against each other, followed by Pearson correlation coefficient and *P*-value calculations.

### Ribo-STAMP bulk RNA sequencing library preparation and analysis

RNA isolation was performed from *n* = 3 biological replicates using Direct-zol RNA microprep kit (ZYMO; R2062). RNA quality was measured through RNA ScreenTape Analysis (Agilent) before proceeding to library preparation. Illumina TruSeq Stranded mRNA kit (Illumina; 20020595) was used for library preparations. Sequencing was performed at the Institute for Genomic Medicine at UCSD on an Illumina NovaSeq. Thirty million single-end reads were sequenced per sample. FASTQ files were trimmed for indices using cutadapt 1.18. Trimmed FASTQ files were subsequently aligned to the mm10 genome using the STAR alignment software v.2.5.2b, with the flags --outSAMAttributes All, --outFilterMultimapNmax 10, --outFilterMultimapScoreRange 1, --outFilterScoreMin 10, --alignEndsType EndToEnd. BAM files were processed using the SAILOR^71^ pipeline to derive the number of edits per gene. Using a custom in-house pipeline, BAM files were processed using feature counts v.1.5.3 to assess the number of reads aligned to each gene. Both edits per kilobase per million mapped reads (EPKM) and EPR values were calculated for each gene for each sample, using edit counts and read counts derived previously.

### Differential transcription versus translation analysis after BDNF

DESeq2 v.1.39.3 was used to calculate RNA log_2_FC and *P* values for the comparisons between the BDNF 15′ and no-treatment and BDNF 60′ and no-treatment conditions. A two-sided *t*-test was used to compare the three replicates of BDNF 15′ and BDNF 60′ EPR values for each gene with the three replicate values of the no-treatment condition. The log_2_FC values were calculated as the log_2_ of the mean of the BDNF 60′ or 15′ EPR values, respectively, minus the log_2_ of the mean of the no-treatment EPR values. *P* values were adjusted for multiple hypothesis testing using the Benjamini–Hochberg correction. Dox-induced genes shown in Extended Data Fig. 1c were removed from the analyses. IEGs known to be BDNF-induced and activity-induced were taken from previous studies^72,73^. Previously validated BDNF-induced transcripts were taken from published literature^64,74–77^.

### Gene Ontology analyses

Gene Ontology analyses were performed using clusterProfiler v.4.10.1 (ref. ^78^) in R studio, with the exception of that shown in Fig. 2. For comparisons among gene lists, the compareCluster function was used with fun = enrichGO and pAdjustMethod = BH (Benjamin–Hochberg) and with pvalueCutoff = 0.01 and qvalueCutoff = 0.05. Redundant Gene Ontology terms were removed using the ‘simplify’ function with standard settings and cutoff = 0.7. For CA3 EPR up genes in Fig. 6g, the enrichGO function was used with the same settings as above. All genes detected by scRNA-seq in the cell types subjected to a specific analysis were used as background. Biological Process ontologies were visualized unless otherwise indicated. Identification of enriched synaptic terms from ‘EPR up’ and ‘EPR not up’ genes in Fig. 2g was executed using SynGo^79^, using the ‘brain expressed’ background Gene Set Enrichment Analysis setting of ‘min. gene count per term’ set to five and considering the function (biological processes) readout. The lists of ‘EPR up’ and ‘EPR not up’ unique presynaptic and postsynaptic genes used in Fig. 2i–k were manually extracted on the basis of the presynaptic or postsynaptic ontology terms associated with the genes by SynGO. All synaptic genes in Fig. 2k correspond to all the genes retained by SynGo for analysis in the respective conditions. Dox-induced genes shown in Extended Data Fig. 1c were removed from the analyses.

### Stereotactic surgery and single-cell dissociation

Single-cell suspensions from hippocampi were obtained following Hochgerner et al.^80^. Three male P22 C57BL/6J mice were stereotactically injected in the dorsal hippocampus with 800-nl EF1a-Ribo-STAMP virus (2.77 × 10^13^ viral genomes ml^−1^) bilaterally using the following coordinates (from bregma): anterior–posterior, −2 mm; medial–lateral, ±1.7 mm; and dorsal–ventral, −1.6 mm. All mice were processed separately to maintain independent replicates. P25 mice were anaesthetized 72 h later and intracardially perfused with cold carboxygenated artificial cerebrospinal fluid. Brains were sliced in 300-μm-thick coronal sections. Dorsal and dorso-ventral hippocampi were manually microdissected, incubated in Papain/DNaseI (Worthington dissociation kit) and mechanically dissociated. The resulting single-cell solutions were filtered, centrifuged through a 5% OptiPrep gradient and diluted to a final concentration of 1,200 cells µl^−1^ for scRNA-seq processing.

### Single-cell library preparation and analysis

Single-cell libraries were constructed using the 10x Genomics Chromium Next GEM Single Cell 3′ Reagent Kits v.3.4. The target cell recovery was 10,000 cells per replicate. Single-cell libraries were sequenced at the Genomics Core at The Scripps Research Institute on an Illumina NextSeq200 with a target of 100,000 reads per cell. The 10x FASTQ files were aligned with Cell Ranger v.3.0.0 using a reference transcriptome modified to include mm10 genome and the tdTomato sequence from the Ribo-STAMP construct. Scrublet was used to remove doublets in the data^81^. Scanpy was used for single-cell analysis and plotting (https://scanpy.readthedocs.io/en/stable/). Quality control was performed separately on each replicate, as recommended in the Scanpy documentation. All replicates were filtered to remove genes in which fewer than five cells had expression values greater than 0, cells with less than 2,000 genes expressed, cells with more than 10,000 genes expressed and cells with more than five mitochondrial counts. The following filters were used to filter cells on the basis of *Gm42418* counts: rep1, *Gm42418* less than 7.5; rep2, *Gm42418* less than 10; and rep3, *Gm42418* less than 15. Counts per million (CPM) and log normalization were used for RNA counts. Batch balanced *k*-nearest neighbours batch correction was performed on samples using Scanpy^82^. Cell assignments were done using RNA counts using known marker genes^14,83,84^.

### Single-cell and isoform-level edit calling

#### Overview

MARINE is a versatile, robust, memory-efficient and ultra-fast Python package we developed to identify RNA edits at single-cell and isoform-level resolution in our dataset (Supplementary Fig. 2a). The modular framework of MARINE includes components for read processing, edit identification and coverage calculation that can be leveraged for both bulk and single-cell processing, with extra features tailored to single-cell data. MARINE is implemented in Python, leveraging pysam for alignment manipulation, pandas and polars for data handling and multiprocessing for parallelization.

#### Inputs

Any aligned.bam file, whether derived from single-cell or bulk sequencing libraries and using either single-end or paired-end and either short or long reads, can in principle be analysed using MARINE. For single-cell data, a list of valid cell barcodes can be provided as a whitelist, ensuring that only reads associated with recognized cells are included in the analysis. We analysed only the cells not removed during the quality-control filtering step.

#### Edit calling and filtering

To optimize parallelization in the edit identification step, the input.bam file is divided into smaller regions defined by genomic contigs and intervals, with each region processed independently. MARINE simultaneously tabulates all edit types at all non-reference positions in each cell (or cell–isoform combination) by combining aligned read sequence information with information derived from the mismatch descriptor (MD) and Compact Idiosyncratic Gapped Alignment Report (CIGAR) read tags. Reads are excluded if they fail quality control checks, are unmapped, are unique molecular identifier duplicates (do not contain the xf:25:i tag), represent secondary or supplementary alignments or do not meet user-specified thresholds for mapping quality. Bases are excluded if they fail user-defined filters for base quality or proximity to read ends, in which base quality is known to drop off.

#### Coverage calculation

MARINE efficiently calculates per-cell coverage information at these positions through reconfiguration of aligned read metadata. Reads are regrouped by chromosome and barcode, and the contig name is modified to include both the genomic region and the cell barcode (for example, chr1_<barcode>). This modification enables downstream tools such as Samtools82 to treat each cell–barcode pair as a unique entity. Coverage at edited sites is then calculated independently for each cell–barcode pair, quantifying reads supporting reference and alternate bases at every genomic position in cells with the edit.

#### Benchmarking for accuracy, speed and memory usage

Although earlier tools addressed edits at single-cell resolution^85^, MARINE can rapidly identify barcode-specific edits de novo. Given *n* edited reads, MARINE operates with a time complexity of O(*n*) (Supplementary Fig. 2b), whereas memory usage can be capped at a constant O(*c*) (Supplementary Fig. 2c). Although MARINE can run efficiently on a single CPU, its performance is significantly enhanced when executed across several cores; for systems with limited RAM, MARINE can be throttled to process fewer chromosomes simultaneously, reducing memory requirements at the cost of increased runtime (Supplementary Fig. 2d). Although primarily designed for single-cell data, MARINE boasts a best-in-class balance of speed and memory efficiency when applied to bulk datasets, with memory and time complexity also of O(*n*) and O(*c*), respectively. Despite minor differences resulting from algorithm-specific filters, MARINE reliably detects all RNA editing sites identified by both REDItools2 and JACUSA2, two widely used multiprocessing-enabled tools for RNA editing detection (Supplementary Fig. 2e). MARINE achieves runtimes comparable to JACUSA2 while maintaining memory usage similar to REDItools2.0 (Supplementary Fig. 2f,g). At its highest efficiency, with increased core usage, MARINE surpasses both tools in speed and memory efficiency (Supplementary Fig. 2f,g).

#### Outputs

Depending on parameters, both coverage and edit data can be aggregated into a sparse matrix format, with cell barcodes as rows and genomic positions as columns, enabling integration with single-cell transcriptomic analysis frameworks, such as Scanpy. Outputs include site-level summaries and, optionally, .bedgraph files for visualization in tools, such as IGV, or BED files formatted for downstream tools, such as FLARE.

### Short-read EPR pipeline for single cells

Edit sites (sites with more than one edit type) and edits overlapping SNPs found in the dbSNP mm10 database were filtered out, as were edit sites with less than three total edits across all cells, to yield edit counts per cell. Edits and Cell Ranger raw counts were used to compute EPR values for each gene within each cell. Edit sites that mapped to antisense reads were determined by strand-specific intersection with an annotation gtf file and subsequently filtered out. In addition, edits at which more than 5% of reads were edited were filtered out. After these filters, 65%, 62% and 63% of edit sites remaining were C>U for the three replicates, respectively.

The EPR levels for each replicate were normalized to Stamp expression using linear regression. Briefly, for each cell, the mean EPR value was calculated as the sum of EPR values across all edited (with more than zero edits and three or more reads) genes, divided by the number of edited genes. For cells with non-zero Stamp expression, we performed ordinary least squares linear regression, using the log-transformed Stamp CPM counts as the independent variable and the log-transformed mean EPR as the dependent variable:

$$\log [{\rm{m}}{\rm{e}}{\rm{a}}{\rm{n}}\,{\rm{E}}{\rm{P}}{\rm{R}}+1]=A\times {\rm{l}}{\rm{o}}{\rm{g}}[{\rm{S}}{\rm{t}}{\rm{a}}{\rm{m}}{\rm{p}}\,{\rm{C}}{\rm{P}}{\rm{M}}+1]+{b}$$

The regression residuals were extracted and exponentiated to reverse the log transformation and then rescaled and shifted to preserve the original dynamic range and avoid negative values. Rescaling was done by aligning the minimum and maximum of the residual distribution to those of the uncorrected mean EPR. Translation ensured that the minimum corrected EPR matched the original minimum EPR. These regression residuals represent the Stamp-corrected EPR values.

A cell-specific normalization factor was then calculated as the ratio of corrected to original mean EPR. The edit counts of each cell were multiplied by its corresponding factor to produce an adjusted editing matrix. The read count matrix was masked using a minimum read-depth threshold of 5, preserving edit counts only for gene–cell pairs with five or more reads and setting others to zero. Final EPR matrices were recalculated as the ratio of normalized edit counts to unadjusted read counts. Only cells containing at least five edited genes were retained for downstream analysis.

### Clustering cells by EPR

To validate that edits were deposited in a cell-type-specific manner, we clustered cells by EPR and evaluated whether these new clusters matched the RNA-based cell assignments. Clustering single cells by EPR was very noisy and not definitive. We reasoned that the sparsity of edits precluded to identify cell types. Thus, to overcome this limitation, we created pseudocells that represented 5, 10 or 15 cells from the same cell-type assignment (on the basis of RNA) randomly grouped. We summed the normalized edit counts and raw read counts. We then calculated EPR as edits/reads for each pseudocell. We repeated this five times to account for variation in the shuffling to create five randomly shuffled EPR datasets. Log normalization, data scaling, principal component analysis, neighbour calculations, UMAP and Leiden clustering were performed. We plotted this in UMAP space for both EPR Leiden clusters and the original cell-type assignments. For cell types with the largest numbers of cells, clustering by EPR replicated the original RNA cell assignments, and the more cells included (15 cells), the clearest clusters separated. Representative UMAPs from one shuffle replicate are shown in Extended Data Fig. 5. All five shuffle replicates yielded similar results.

### Differential EPR analysis across all cell types

Differential expression analysis was done for EPR using Scanpy’s rank gene groups with groups=‘all’, reference=‘rest’ and method=‘wilcoxon’ to find EPR marker genes (Fig. 3e). For each annotated cell type, genes with a positive log-fold change and *P* < 1 were considered. To ensure specificity, we excluded genes that were also significantly expressed (*P* < 0.05) in any other cell type. The remaining genes were sorted by their ranking score, and the top 3 genes per cell type were selected for dot-plot visualization. For Gene Ontology enrichment analysis, the top 100 genes by ranking score were used per cell type.

### EPR relationship to RNA

#### Cell-type analysis

Pseudobulk EPR and RNA counts were calculated before correlations and clustering. To compute pseudobulk EPR and RNA counts, we processed single-cell data grouped by cell type and sample. A minimum raw read threshold of 5 was applied to filter low-coverage gene–cell observations. For each unique combination of cell type and sample, total EPR was calculated across cells as the sum of edits divided by the sum of raw reads per gene. The mean of total EPR was then calculated across samples per cell type for downstream analysis. All cell types were merged into one dataframe. Next, we applied a multistep filtering procedure to remove genes with low reads or edits. For each gene, we required that at least one cell type exhibited sufficient coverage across all samples. Specifically, we retained genes when (1) total reads were five or more in all samples of at least one cell type; (2) total edits were five or more in all samples of at least one cell type; and (3) at least one cell per sample passed the read threshold in at least one cell type. These conditions were applied to a multi-indexed dataframe containing pseudobulk metrics per gene, cell type and sample. The final filtered matrix included only genes passing all three criteria. For each unique combination of cell type and sample, total counts were calculated across cells as sum of normalized counts per gene, and mean counts were calculated across cells as mean of normalized counts. Normalized counts refer to CPM and log normalization. The mean of total counts (total normalized counts) and mean of mean counts (mean normalized counts) were then calculated across samples per cell type for downstream analysis. Spearman correlation was performed between pseudobulk EPR and pseudobulk total normalized RNA counts and visualized using a seaborn.heatmap() (Fig. 3f). To visualize EPR of all genes across cell types (Extended Data Fig. 5c,d), we used seaborn.clustermap() with settings standard_scale=‘row’ and metric=‘braycurtis’ for pseudobulk EPR. To visualize RNA counts in the same order as EPR, we used pseudobulk RNA mean normalized counts with settings standard_scale=‘row’ and clustering turned off.

#### Oligodendrocyte analysis

Pseudobulk EPR and RNA normalized total counts for oligodendrocytes were computed as described above. Genes were retained only if they met the following criteria across all samples: (1) total reads of five or more; (2) total edits of five or more; and (3) at least five cells passing the minimum read threshold. To identify and visualize clusters of similarly expressed genes, we applied agglomerative hierarchical clustering to a merged dataset of pseudobulk EPR and RNA normalized total counts. Pairwise distances between rows were computed using the Bray–Curtis dissimilarity metric. Clustering was performed using the AgglomerativeClustering algorithm^86^, with average linkage and a precomputed distance matrix. A manual number of clusters (*n*_clusters = 8) was specified to reflect visual patterns. Clusters were visualized using seaborn.clustermap(). A scale factor of 1,000,000 was multiplied to EPR values to ensure similar scaling next to RNA counts for visualization.

### Translational cell state analysis

#### Identifying translational cell states

CA1.2 and CA1.4 were annotated as high translation and CA1.1 and CA1.3 as low translation manually by using RNA cell assignments. To categorize CA3 cells into high and low translation groups on the basis of editing rate, we used Gaussian mixture modelling. Specifically, we applied Gaussian mixture modelling with two components to the per-cell total editing rate, calculated as the ratio of total edits to total reads. The model was fit using the GaussianMixture implementation from scikit-learn (*n*_components = 2; random_state = 42). Cells were then assigned to one of two clusters on the basis of the maximum posterior probability. The cluster with the higher mean was designated as the high group and the other as the low group. For CA3 filtering, cells were retained between 0.65 and 0.80 quantiles on the basis of Ribo-STAMP RNA counts.

Differential gene expression analysis using scanpy.tl.rank_genes_groups was performed with EPR between high and low groups for CA1 and CA3 to identify genes undergoing translation in the high groups. The statistical method used was ‘Wilcoxon’. For bursty gene analysis, CA1 subfield marker genes^4,87^ and bursty marker genes^88^ were used.

### Differential EPR versus RNA analysis between CA3 and CA1

To identify genes with differential translation between CA3 and CA1 neurons, we analysed EPR across several biological replicates. For each replicate, genes were retained for analysis if they had editing events (EPR > 0) in at least two cells. Cells were retained for each gene if they had a minimum of five raw reads.

For each gene in each replicate, we computed the sum of edits and raw reads across CA3 and CA1 cells, respectively. EPR was then calculated as the total number of edits divided by the total number of raw reads per cell type. For downstream analysis, we included only genes with non-zero EPR values in all three replicates.

Mean EPR was computed across replicates and used for log_2_FC of translation for CA3 compared with CA1 neurons (log_2_[CA3_EPR/CA1_EPR]). To assess statistical significance, we performed vectorized bootstrap resampling (*n* = 10,000 iterations). In each iteration, the EPR values across replicates were resampled with replacement and randomly reassigned to CA3 or CA1 group. The test statistic was the mean EPR difference across conditions. Empirical *P* values were calculated on the basis of the fraction of bootstrap samples with a mean difference greater than or equal to the observed statistic.

For differential count expression analysis between CA3 and CA1, we used scanpy.tl.rank_genes_groups.

For mean EPR of CA3 EPR upregulated ribosomal proteins of the small (RPS) or large (RPL) subunits and translation factors in CA1 and CA3 neurons shown in Fig. 6h, the mean EPR across replicates was used.

### CA3/CA1 Ribo-STAMP correlations to existing datasets

The ratio of CA3 EPR to CA1 EPR for each gene was calculated in terms of log_2_FC. Existing datasets were standardized for comparison as follows: (1) For RiboTag^16^, no further processing was necessary. The precalculated CA3/CA1 log_2_FC value was used from this dataset to make our comparisons with EPR. (2) For RIBOmap/STARmap^27^, RTE was calculated as the ratio of RIBOmap and STARmap signals from sequential brain slices across all genes. RTE for each gene was calculated for CA3 and CA1, and then a log_2_FC quantity was calculated for the ratio of CA3 RTE to CA1 RTE. (3) For human hippocampus mass spectrometry dataset^89^, the precalculated log_2_FC of CA1/CA3 grouped abundances was inverted (*−1) to obtain a comparable CA3/CA1 log_2_FC value for each gene. Correlation plots were made in which the *x* axis represents a cell count filter, such that at each location along the axis, only genes expressed in at least that number of cells in both CA1 and CA3 were used to calculate the Spearman correlation coefficient. Additionally, genes were retained for the analysis if EPR changes between CA3 and CA1 yielded *P* < 0.05. The permutation test involved calculating correlations using 100 random shuffles of EPR values within each cell count threshold-derived gene subset.

### Single-cell long-read library preparation and analysis

#### MAS-seq library preparation

Single-cell MAS-seq libraries of single-cell hippocampal RPS2-STAMP complementary DNA (cDNA) generated from the 10x Genomics 3′ kit (10x Genomics; v.3.1; PN-100026) were prepared for long-read single-cell sequencing using the MAS-seq for 10x Single Cell 3′ Kit (Pacific Biosciences; 102-659-600; protocol v.1.03) with the following modifications. To increase library yield while mitigating amplification artefacts, we increased the MAS–PCR volume to 50 µl and the input purified cDNA to 80 ng and decreased the PCR cycle number to seven cycles. Following MAS–PCR, the SMRTbell cleanup bead ratio (Pacific Biosciences; 102-158-300) was altered from 1.5× bead addition volume to reaction volume to 1.35×. Finally, post-array ligation, post-DNA damage repair and final cleanup were purified with a 1.55× SMRTbead cleanup. Following MAS library construction, libraries were quantified with a Qubit dsDNA High Sensitivity Assay Kit (Thermo Fisher Scientific; Q32854) and a Femto Pulse (Agilent; FP-1002-0275). Libraries were sequenced on one 25M SMRT cell (102-202-200) on the Revio platform of Pacific Bioscience.

#### MAS-seq data processing

The raw HiFi reads generated had average read lengths around 14 kb across 16-mer MAS arrays for all replicates. HiFi reads are circular consensus reads with 90% of bases having Q30 or higher and a median read accuracy of Q30 or higher. These HiFi reads were processed to extract de-concatenated or segmented reads (S-reads) representing the original cDNA sequences, using the SKERA tool of PacBio (https://skera.how/).

Reads were processed in accordance with the PacBio single-cell workflow (https://isoseq.how/umi/high-level-workflow.html) to obtain reads with corrected cell barcode (CB) tags. Deduplication was completed using isoseq groupdedup (v.4.2.0) with default parameters. Reads were filtered to include only those with barcodes assigned to a cell type in the short-read data using samtools view (v.1.18) with parameters -h -D CB. Note that the long-read sequencing barcodes are the reverse complement of the short-read sequencing barcodes. Pbmm2 align (v.1.16.1) was then used to align the reads using the Cell Ranger mm10 reference annotation with the parameter --sort. Samtools (v.1.18) calmd was used to add mismatch descriptor tag. The mismatch descriptor tag is a tag contained within the aligned BAM file that contains information regarding mismatched and deleted reference bases. We use this tag to call edits. Samtools view (v.1.18) was used to filter reads to include only primary alignment reads using the parameter -F 2316.

Reads were assigned to annotated isoforms and their expression quantified using IsoQuant^85^ (v.3.3.0) with parameters --data-type pacbio, --transcript_quantification unique_only and --gene_quantification unique_only. The read assignment output from IsoQuant, together with a custom script, was used to add isoform tags ‘IB’ and ‘IS’ for each read, indicating the isoform to which it was assigned. For this proof-of-concept study, we elected to focus only on annotated isoforms to ensure reliable results.

#### Long-read edit calling and analysis

MARINE was used to find C>T edit loci for each read. Subsequently, C>T edits were aggregated by isoform to assess isoform-specific edits. Edited sites were removed if they were found among annotated mm10 SNPs obtained from dbSNP. Cell types were assigned on the basis of cell-type assignments completed with short-read data. A matrix of read counts and edits for each isoform and each cell was then generated using the read assignments and called edits, respectively, and used for downstream analysis. We quantified editing for each isoform within single cells using the EditsC metric—the ratio of edited cytosines (C-to-U) to total cytosines across all exons and raw reads:

$$\mathrm{EditsC}=\mathrm{total}\,\mathrm{edited}\,\mathrm{Cs}/(\mathrm{total}\,\mathrm{Cs}\,\mathrm{in}\,\mathrm{transcript}\,\mathrm{annotation}\,\,\times \,\mathrm{total}\,\mathrm{reads})$$

Cells were excluded if they contained five or fewer edited genes, and transcripts were excluded if five or fewer cells contained edits more than 0. EditsC was normalized to Ribo-STAMP expression following the same approach as outlined previously for short read. Correlation between short-read EPR and long-read EditsC was done by calculating the sum of EPR and the sum of EditsC using overlapping genes with a minimum total count of 1,000 across replicates. CPM was calculated, and log normalization was performed on RNA counts. Scanpy was used for single-cell analysis (https://scanpy.readthedocs.io/en/stable/). We referred to isoforms using Cell Ranger mm10 gtf annotation.

### Long-read pseudobulk EditsC and RNA generation

For each cell type, EditsC was calculated as described above. However, edits and reads were summed across cells for each sample. Mean EditsC was calculated across samples and used for heat-map visualization in Fig. 5b and Extended Data Fig. 9a. Normalized RNA counts were summed across cells for each sample. Mean normalized RNA counts were calculated across samples and used for heat-map visualization in Extended Data Fig. 9b.

### Cell-type-specific EditsC and RNA analysis

Pseudobulk datasets generated from above were used. Genes were filtered for a minimum of one read and one edit per sample for at least one cell type. Only transcripts with more than one isoform per gene were used in the analysis. Heat maps were made using clustermap from seaborn^90^, with settings standard_scale=‘row’, col_cluster=False and metric=‘braycurtis’.

### EditsC and RNA isoform correlations

To evaluate coordination of transcription and translation across transcript isoforms, we computed pairwise Spearman correlations using pseudobulk EditsC and normalized RNA counts from above. For genes with two or more isoforms, we calculated pairwise Spearman correlations between their mean values across cell types. The results were visualized using seaborn.heatmap(). To explore transcript features associated with discordant translation, we identified isoform pairs with non-positive Spearman correlations (≤0) from the isoform EditsC correlation matrix. For each pair, transcript biotypes were annotated using the Cell Ranger-mm10-3.0.0 GTF.

### Quartile analysis

For each cell-type pseudobulk dataset, we filtered genes to retain only transcripts with non-zero edits across all samples. For each filtered dataset, we used stratified transcripts into quartiles using pandas.qcut, assigning labels from Q1 (lowest) to Q4 (highest). Transcript names from Q1 and Q4 were used for downstream transcript features analysis.

### High versus low translation of isoforms from the same gene

For each cell-type pseudobulk dataset, we filtered genes to retain only transcripts with non-zero edits across all samples and when the total read count across all samples was greater than 20. Genes with multiple isoforms were retained for further analysis. Next, we selected the isoforms with the highest and lowest EditsC values to represent the high and low translated isoforms, respectively. High and low transcript names were used for downstream transcript feature analysis. High and low isoforms were visualized by cell type using seaborn.clustermap().

### Transcript feature analysis

To assess the relationship between transcript-level EditsC and features of transcripts, we obtained the coordinates for 5′ UTRs and 3′ UTRs Cell Ranger mm10 annotation.

### GC content and motif analysis

To conduct a motif analysis for transcripts of each designated cell type and group, the 5′ UTR, CDS and 3′ UTR coordinates were used to obtain associated sequences from the Cell Ranger mm10 reference. Using these sequences, GC content was calculated and a FASTA file was generated for each group, which was then used as input to MEME v.5.3.0 (ref. ^91^) to obtain enriched motifs.

### miRNA analysis

Annotated miRNA binding regions associated with the mm10 reference were obtained from TargetScanMouse (release 8.0) (refs. ^92,93^). Using pybedtools (v.0.9.1), to overlap 3′ UTR coordinates and miRNA target regions, we obtained the number of miRNA binding sites associated with each transcript. The differences in number of miRNA target sites associated with transcripts of each group were compared using a two-sided Mann–Whitney *U*-test.

### ELAV binding analysis

Neuronal ELAV eCLIP binding sites identified in at least five of six independent experiments conducted by Ince-Dunn et al.^94^ were lifted over from mm9 to mm10 coordinates using LiftOver from UCSC Genome Browser^95^. The coordinates were then intersected with coordinates of 3′ UTRs of transcripts associated with each group. Significance was calculated using a two-sided Mann–Whitney *U*-test.

### Genome browser tracks

BED files were generated using the SNP-filtered edit information output from MARINE. To analyse isoform-specific editing sites within the *Cadm3* gene, we filtered genomic BED files for two isoforms: *Cadm3-202* (ENSMUST00000111220) and (ENSMUST00000005470). Isoform-specific BED files were then visualized using IGV. Genome browser tracks from Furlanis et al.^16^ were saved from their SPLICECODE database (https://scheiffele-splice.scicore.unibas.ch). Example edit and Furlanis et al.^16^ tracks were aligned with chromosome start and end sites.

### Motif analysis

UTR sequences of each cluster were analysed for enriched motif sequences using MEME v.5.3.0 (ref. ^91^). TomTom v.5.5.5 (ref. ^96^) was used to identify known motifs with strong similarity to those identified in the clusters. Because the presence of multiple gene isoforms could affect the results of the motif analysis, we tested isoform enrichment in each quadrant of Fig. 5f. Of the 769 genes with significant EditsC enrichment (*P* < 0.05), only 18 had two isoforms of the same gene represented (*Zeb2*, *Trove2*, *Tmed7*, *Tmed10*, *Sc5d*, *Rtn3*, *Rnf11*, *Rasal2*, *Qk*, *Nfasc*, *Nedd4*, *Ndfip2*, *Ncam2*, *Msmo1*, *Hnrnpu*, *Ensa*, *Eif4g2* and *Commd6*). Four of the 18 genes (*Rasal2*, *Msmo1*, *Hnrnpu* and *Ensa*) exhibited isoform discordance, with one isoform showing higher EditsC in astrocytes and the other in oligodendrocytes. Further, the isoforms of 5 of 14 genes remaining (*Trove2*, *Tmed10*, *Ndfip2*, *Eif4g2* and *Commd6*) were not in the same quadrant. Thus, we conclude that it is very unlikely that the results of motif analysis were driven by multiple isoforms of the same gene in the same quadrant.

### Differential transcript analysis

To evaluate differential translation dynamics between cell types, pairwise comparisons were performed between all cell types. For each cell type, pseudobulk EditsC per sample were used from above. Transcripts with zero reads and zero edited counts across any sample were excluded. For each transcript, the mean EditsC was calculated per cell type, and log_2_FC was computed to quantify editing differences. A two-sample unequal variance *t*-test was applied to EditsC values between the two cell types across samples for statistical comparison. The oligodendrocyte versus astrocyte comparison yielded the greatest number of significant transcripts and was therefore used for further analysis. For differential RNA expression analysis, scanpy.tl.rank_genes_groups was used with method = ‘wilcoxon’. CA3 compared with CA1 was used for Fig. 6 and Extended Data Fig. 10. Discordant translated isoforms from the same gene were identified by (1) filtering for transcripts in which *P* < 0.05; (2) grouping transcripts by gene; and (3) identifying genes in which at least one isoform had increased EditsC (positive log_2_FC) and another had decreased EditsC (negative log_2_FC).

### Animal experiments

No statistical methods were used to predetermine sample sizes. For mice experiments, we based our numbers on previously published studies and used at least three biological replicates for each experiment. Mice were randomly assigned into experimental groups. Experiments were not blinded. For CA1 and CA3 protein expression analyses, blinding was not possible because pictures were taken from the same brain slices.

### Statistics

Measurements were taken from distinct samples, as indicated in the figure legends. Adjustment and correction methods are reported in text and figure legends, when applicable. EPR and EditsC analyses were performed without adjustments or corrections.

### Reporting summary

Further information on research design is available in the Nature Portfolio Reporting Summary linked to this article.

## Online content

Any methods, additional references, Nature Portfolio reporting summaries, source data, extended data, supplementary information, acknowledgements, peer review information; details of author contributions and competing interests; and statements of data and code availability are available at 10.1038/s41586-026-10118-1.

## Supplementary information

Supplementary Figures Supplementary Figs. 1 and 2.

Reporting Summary

Supplementary Table 1 RNA differential expression analyses comparing 48-h and no-Dox neurons in Extended Data Fig. 1c.

Supplementary Table 2 Dataset from Fig. 2c and Extended Data Fig. 2a,b. EPR and RNA differential expression analyses comparing BDNF versus no-treatment neurons and raw data from select genes.

Supplementary Table 3 SynGO analysis table from Fig. 2.

Supplementary Table 4 Gene Ontology analysis of cell-type-specific EPR from Extended Data Fig. 5c,d.

Supplementary Table 5 EPR and RNA differential expression and Gene Ontology analysis on CA1 and CA3 high and low translation clusters from Fig. 4 and Extended Data Figs. 6 and 7.

Supplementary Table 6 Statistics for transcript feature analysis in Fig. 5c and Extended Data Fig. 9f,g.

Supplementary Table 7 Example isoforms validated in RiboTag dataset^16^.

Supplementary Table 8 EditsC pairwise comparisons between cell-type pairs.

Supplementary Table 9 EditsC and RNA differential expression analysis in oligodendrocytes versus astrocytes.

Supplementary Table 10 Datasets used in Fig. 6 and Extended Data Fig. 10: short-read EPR and RNA differential expression analyses, long-read EditC and RNA differential expression analysis and RIBOmap/STARmap analysis.

Supplementary Table 11 Gene Ontology term analysis from Fig. 6g.

Supplementary Table 12 Canonical 5′TOP mRNA list used in Extended Data Fig. 10i.

## Data Availability

Differential expression and translation and Gene Ontology analyses are available as Supplementary Tables 1–12. All raw and processed bulk, single-cell and long-read RNA-seq files are deposited at Gene Expression Omnibus under accession no. GSE314176. Other datasets used in this study include the RiboTag mouse hippocampus dataset (https://www.nature.com/articles/s41593-019-0465-5)^16^, RIBOmap dataset (https://www.science.org/doi/10.1126/science.add3067)^27^ and human hippocampus dataset (https://pubs.acs.org/doi/10.1021/acs.jproteome.2c00143)^89^.

## Code availability

MARINE is freely available at https://github.com/YeoLab/MARINE, with comprehensive documentation, example scripts and integrated tests to ensure code reliability and encourage open-source contributions.
