# Supplementary material for: Single-cell and isoform-specific translational profiling of the mouse brain
Source: Nature. 2026 Feb 18;652(8111):965–77. doi: 10.1038/s41586-026-10118-1 (PMC13102718; doi:10.1038/s41586-026-10118-1)
Supplement: Supplementary file 1 — Supplementary Figs. 1 and 2. [file 41586_2026_10118_MOESM1_ESM.pdf]

nature portfolio

https://doi.org/10.1038/s41586-026-10118-1

Supplementary information

Single-cell and isoform-specific translational profiling of the mouse brain

In the format provided by the authors and unedited

a

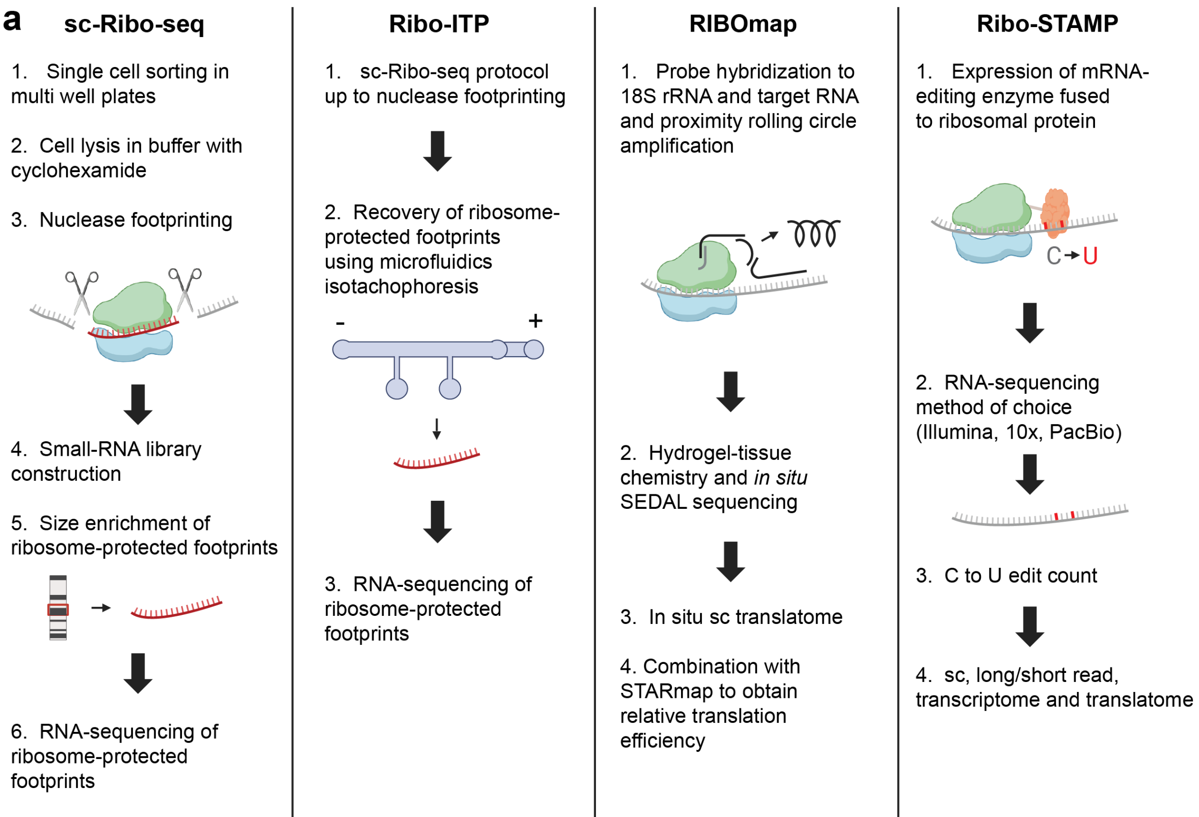

	sc-Ribo-seq	Ribo-ITP	RIBOmap	Ribo-STAMP
**ADVANTAGES**	• Correlates with Ribo-seq	• Correlates with Ribo-seq<br>• Optimized for low input samples, high RNA yield<br>• Faster protocol than sc-Ribo-seq<br>• Automated	• Correlates with RiboLace<br>• Can investigate a lot of cells at once (50,000)<br>• Enables spatial information within tissue<br>• Enables subcellular spatial information in cultured cells	• Correlates with Ribo-seq, polysome profiling, and Ribo-eCLIP<br>• No need for custom equipment or technical know-how<br>• Can be coupled with standard sequencing methods (Illumina, 10x, PacBio), enabling isoform measurements.<br>• Transcriptomics and translatomics with same RNA molecules
**DISADVANTAGES**	• Complicated protocol (FACS, nuclease digestion, small RNA library prep, size selection)<br>• Loss of input material, not ideal for low input samples i.e. brain cells<br>• Low RNA yield<br>• Lacks isoforms resolution<br>• Requires transcriptomics and Ribo-seq in different cells, not a direct readout of translation efficiency<br>• Limited by sequencing depth	• Complicated protocol (Similar to Ribo-seq)<br>• Requires custom equipment and microfluidics know-how)<br>• Lacks isoforms resolution<br>• Requires transcriptomics and Ribo-seq in different cells, not a direct readout of translation efficiency<br>• Limited by sequencing depth	• Complicated protocol (custom probe design, requires cell segmentation)<br>• Requires microscope, microfluidics, and technical know-how<br>• Limited by pre-defined gene set of low to medium expressed genes and probe design<br>• Lacks isoforms resolution<br>• Requires STARmap and RIBOmap in different samples - not a direct readout of translation efficiency	• Limited by sequencing depth and enzyme editing efficiency

Supplementary Information Fig.1. Schematic of sc translational profiling techniques. a, Schematic (top), advantages (middle), and disadvantages (bottom) of the four sc translational profiling technologies (sc-Ribo-seq, Ribo-ITP, RIBOmap, and Ribo-STAMP). Briefly, scRibo-seq

is restricted by loss of input material, Ribo-ITP is better suited for low cell numbers but requires custom microfluidics, and RIBOmap requires a pre-selected list of genes for analysis, hindering unbiased discovery. Figure created in BioRender.

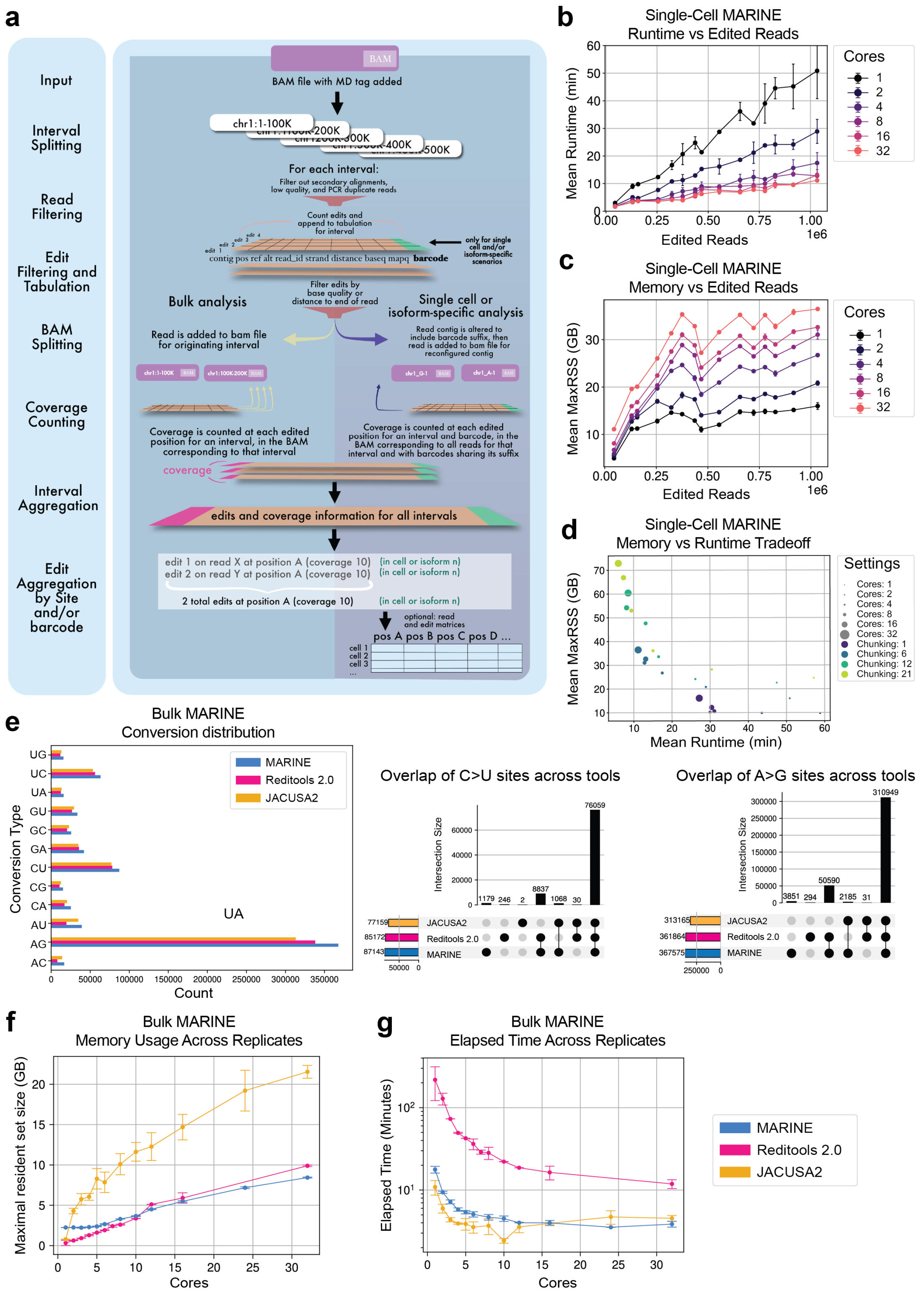

Supplementary Information Fig. 2. MARINE is a new computational tool that enables efficient analysis of RNA editing at single-cell resolution. a, Flowchart depicting MARINE's workflow for calling RNA edits of all types in bulk and single-cell samples. **b**, MARINE runs on

single-cell data with O(n) time complexity, and runs faster with more cores. **c**, MARINE runs on single-cell data with O(c) memory complexity, depending on RAM available. **d**, By throttling the number of contigs simultaneously processed, users can find an optimal trade-off between memory and runtime for their runtime environment. **e**, Edit conversion distributions from MARINE, Reditools2.0 and JACUSA2 are comparable, with concordant site discovery across all three tools. **f**, MARINE runs on bulk data with O(n) time complexity with regard to edited reads; example runs using 32 cores. **g**, MARINE runs on bulk data in with O(c) memory complexity, which can be adjusted by modifying processed genomic interval size; example runs using 32 cores. (**b,f,g**) bars represent standard deviation.
