# Supplementary material for: Single-cell and isoform-specific translational profiling of the mouse brain
Source: Nature. 2026 Feb 18;652(8111):965–77. doi: 10.1038/s41586-026-10118-1 (PMC13102718; doi:10.1038/s41586-026-10118-1)
Supplement: Supplementary file 2 — Reporting Summary [file 41586_2026_10118_MOESM2_ESM.pdf]

Corresponding author(s): Gene W Yeo, Giordano Lippi

Last updated by author(s): 2025/12/15

Reporting Summary

Nature Portfolio wishes to improve the reproducibility of the work that we publish. This form provides structure for consistency and transparency in reporting. For further information on Nature Portfolio policies, see our Editorial Policies and the Editorial Policy Checklist.

Statistics

For all statistical analyses, confirm that the following items are present in the figure legend, table legend, main text, or Methods section.

n/a	Confirmed	
☐	☒	The exact sample size (n) for each experimental group/condition, given as a discrete number and unit of measurement
☐	☒	A statement on whether measurements were taken from distinct samples or whether the same sample was measured repeatedly
☐	☒	The statistical test(s) used AND whether they are one- or two-sided<br>*Only common tests should be described solely by name; describe more complex techniques in the Methods section.*
☐	☒	A description of all covariates tested
☐	☒	A description of any assumptions or corrections, such as tests of normality and adjustment for multiple comparisons
☐	☒	A full description of the statistical parameters including central tendency (e.g. means) or other basic estimates (e.g. regression coefficient) AND variation (e.g. standard deviation) or associated estimates of uncertainty (e.g. confidence intervals)
☐	☒	For null hypothesis testing, the test statistic (e.g. F, t, r) with confidence intervals, effect sizes, degrees of freedom and P value noted<br>*Give P values as exact values whenever suitable.*
☒	☐	For Bayesian analysis, information on the choice of priors and Markov chain Monte Carlo settings
☒	☐	For hierarchical and complex designs, identification of the appropriate level for tests and full reporting of outcomes
☐	☒	Estimates of effect sizes (e.g. Cohen's d, Pearson's r), indicating how they were calculated

Our web collection on statistics for biologists contains articles on many of the points above.

Software and code

Policy information about availability of computer code

Data collection	Bulk RNA Sequencing was performed at the Institute for Genomic Medicine at UCSD on an Illumina Novaseq.<br>Single cell libraries were constructed using the 10x genomics Chromium Next GEM Single Cell 3′ Reagent Kits v3.4.Single cell libraries were sequenced at the Genomics Core at The Scripps Research Institute on an Illumina Nextseq200.<br>Single Cell MAS-Seq libraries of single-cell hippocampal RPS2-STAMP cDNA generated from the 10x Genomics 3’ kit (10x Genomics, v3.1, PN-100026) were prepared for long-read single cell sequencing using the MAS-Seq for 10x Single Cell 3’ Kit (Pacific Biosciences, Cat # 102-659-600, protocol Version 1.03). Libraries were sequenced on one 25M SMRT cell (PN: 102-202-200) on Pacific Bioscience’s Revio platform.<br>Confocal microscopy images were acquired on a Nikon Instruments A1 confocal laser microscope.

Data analysis

RNAseq datasets: analyses, statistics and plots were generated with Python (v3.9.12) and RStudio (v 2023.12.0+369) using the following packages: Short read sequencing: cutadapt (v1.18), STAR alignment software (v2.5.2b), feature counts (v.1.5.3), Deseq2 (v1.39.3), cellranger (v8.0.0), Scanpy (v1.10.3), Numpy (1.26.3), anndata (v0.10.9), pandas (v1.5.3), numpy (v1.26.4), scipy (v1.11.4), matplotlib (v3.9.2), seaborn (v0.13.2), SAILOR (v1.0), MARINE (v1.0.2).
Long read sequencing: PacBio Skera and Single-cell workflow tools, using following packages: isoseq groupdedup (v4.2.0), samtools view (v1.18), Pbmm2 align (v1.16.1), Samtools (v1.18) calmd, IsoQuant (v3.3.0).
Gene Ontology analyses were performed using clusterProfiler (v4.10.1) and SynGo (v1.2).
Confocal imaging analysis: Fluorescence intensity was quantified with Fiji (v2.14.0/1.54f). Percentage of Ribo-STAMP over NeuN-positive neurons was quantified with Cell Profiler (v3.1.9). Statistical tests and plots were generated with GraphPad Prism (v10.2).
MARINE https://github.com/YeoLab/MARINE
SAILOR https://github.com/YeoLab/sailor

For manuscripts utilizing custom algorithms or software that are central to the research but not yet described in published literature, software must be made available to editors and reviewers. We strongly encourage code deposition in a community repository (e.g. GitHub). See the Nature Portfolio guidelines for submitting code & software for further information.

Data

Policy information about availability of data

All manuscripts must include a data availability statement. This statement should provide the following information, where applicable:

- Accession codes, unique identifiers, or web links for publicly available datasets
- A description of any restrictions on data availability
- For clinical datasets or third party data, please ensure that the statement adheres to our policy

Differential expression and translation analyses and GO analyses are available as tables in Supplementary information. All raw and processed bulk-, sc-, and long-read-RNA-seq sequencing files developed for this manuscript are deposited at Gene Expression Omnibus Accession GSE314176.
Ribo-tag mouse hippocampus dataset https://www.nature.com/articles/s41593-019-0465-5
RIBOmap dataset https://www.science.org/doi/10.1126/science.add3067
Human hippocampus dataset https://pubs.acs.org/doi/10.1021/acs.jproteome.2c00143

Research involving human participants, their data, or biological material

Policy information about studies with human participants or human data. See also policy information about sex, gender (identity/presentation), and sexual orientation and race, ethnicity and racism.

Reporting on sex and gender	N/A
Reporting on race, ethnicity, or other socially relevant groupings	N/A
Population characteristics	N/A
Recruitment	N/A
Ethics oversight	N/A

Note that full information on the approval of the study protocol must also be provided in the manuscript.

Field-specific reporting

Please select the one below that is the best fit for your research. If you are not sure, read the appropriate sections before making your selection.

☒ Life sciences ☐ Behavioural & social sciences ☐ Ecological, evolutionary & environmental sciences

For a reference copy of the document with all sections, see nature.com/documents/nr-reporting-summary-flat.pdf

Life sciences study design

All studies must disclose on these points even when the disclosure is negative.

Sample size	No statistical methods were used to predetermine sample sizes. For mice and primary neuronal cultures experiments, we based our numbers on previously published studies and used at least three biological replicates for each experiment unless stated otherwise.
Data exclusions	No data were excluded.
Replication	Experiments were reliably reproduced. Sample sizes are indicated in the figure legends.
Randomization	Mice and cell culture conditions were randomly assigned into experimental groups.

Blinding | Experiments were not blinded because. For CA1 and CA3 protein expression analyses blinding was not possible as pictures were taken from the same brain slices.

Reporting for specific materials, systems and methods

We require information from authors about some types of materials, experimental systems and methods used in many studies. Here, indicate whether each material, system or method listed is relevant to your study. If you are not sure if a list item applies to your research, read the appropriate section before selecting a response.

Materials & experimental systems

n/a	Involved in the study
☐	☒ Antibodies
☐	☒ Eukaryotic cell lines
☒	☐ Palaeontology and archaeology
☐	☒ Animals and other organisms
☒	☐ Clinical data
☒	☐ Dual use research of concern
☒	☐ Plants

Methods

n/a	Involved in the study
☒	☐ ChIP-seq
☒	☐ Flow cytometry
☒	☐ MRI-based neuroimaging

Antibodies

Antibodies used

Primary antibodies:
Rabbit anti-HA (Cell signaling, 3724S, 1:1000 on primary cultures, 1:500 on slices)
Rabbit anti-RPS2 (Bethyl #A303-1054794A, 5 ugs were used for CLIP)
Mouse anti-Puromycin (Kerafast, EQ0001,1:3000)
Rabbit anti-CAMK2α (Abcam, AB52476, 1:5000)
Rabbit anti-SNAP25 (Sigma Aldrich, S9684, 1:1000)
Rabbit anti-STX1A (Synaptic Systems, 110118, 1:500
Rabbit anti-SHANK3 (Synaptic Systems, 162302, 1:500)
Rabbit anti-GRIP1 (Synaptic Systems, 151003, 1:500)
Rabbit anti-PMM2 (Proteintech, 10666-1-AP, 1:500)
Guinea pig anti-MAP2 (Synaptic Systems, 188004, 1:2000)
Guinea pig anti-NeuN (Synaptic Systems, 266004, 1:500)
Mouse anti-GAD67 (Millipore Sigma, mab5406, 1:500)
Rabbit anti-eIF4H (Cell signaling, 3469T, 1:250)
Rabbit anti-eEF2 (Proteintech, 14491-1-AP1, 1:500)
Rabbit anti-RPL6 (Novus Biological, NBP2-20216, 1:500)
Rabbit anti-RPS7 (Proteintech, 14491-1-AP, 1:500)
Rabbit anti-Phospho-eIF2α (Ser51) (Cell signaling, 3398T, 1:250)
Rabbit anti-total eIF2α (Cell signaling , 5324T, 1:500)

Secondary antibodies:
Goat anti-Guinea Pig IgG (H+L) Highly Cross-Adsorbed Secondary Antibody, Alexa Fluor™ 488 (Invitrogen, A11073)
Goat anti-Rabbit IgG (H+L) Highly Cross-Adsorbed Secondary Antibody, Alexa Fluor™ 647 (Invitrogen, A21245)
Goat anti-Mouse IgG (H+L) Cross-Adsorbed Secondary Antibody, Alexa Fluor™ 568 (Invitrogen, A11004).

Validation

Antibodies have been validated with knock down (KD) and knock out (KO) models, by the manufacturers as stated on their websites. Validation statements found in website are reported here following antibody information. When validation statement were not found in the provider's website, example papers with validation are reported where possible.

Rabbit anti-HA (Cell signaling, 3724S, 1:1000 on primary cultures, 1:500 on slices) - Expected to react with all species, application for IHC.

Rabbit anti-RPS2 (Bethyl #A303-1054794A, 5 ugs were used for CLIP) - Reacts with mouse, application for IP. The antibody was validate using ENCODE eCLIP standards.

Mouse anti-Puromycin (Kerafast, EQ0001,1:3000) - Used in seminal paper describing PLA method, 'Direct visualization of identified and newly synthesized proteins in situ'.

Rabbit anti-CAMK2α (Abcam, AB52476, 1:5000) - Anti-CaMKII antibody (ab52476) is a rabbit monoclonal antibody detecting CaMKII in Western Blot, IHC-P, ICC/IF. Suitable for Mouse.For KD validation see 'Dihydrolipoic Acid Inhibits Lysosomal Rupture and NLRP3 Through Lysosome-Associated Membrane Protein-1/Calcium/Calmodulin-Dependent Protein Kinase II/TAK1 Pathways After Subarachnoid Hemorrhage in Rat'

Rabbit anti-SNAP25 (Sigma Aldrich, S9684, 1:1000) - Anti-SNAP-25 recognizes mouse SNAP-25 (25 kD). Applications include the detection and localization of SNAP-25 (25 kDa) by immunoblotting and immunohistochemistry. Staining of SNAP-25 in immunoblotting is specifically inhibited with SNAP-25 immunizing peptide (SNAP-25, mouse, amino acids 9-29 with C-terminally

added lysine).

Rabbit anti-STX1A (Synaptic Systems, 110118, 1:500 - Reacts with mouse. Specific for syntaxin 1A, no cross-reactivity to syntaxin 1B K.O. validated.

Rabbit anti-SHANK3 (Synaptic Systems, 162302, 1:500) - Reacts with mouse. K.O. validated

Rabbit anti-GRIP1 (Synaptic Systems, 151003, 1:500) - Reacts with mouse. Recognizes GRIP 1 and GRIP 2.

Rabbit anti-PMM2 (Proteintech, 10666-1-AP, 1:500) - Tested reactivity: mouse. KD/KO Validated

Guinea pig anti-MAP2 (Synaptic Systems, 188004, 1:2000) - Tested reactivity: mouse.

Guinea pig anti-NeuN (Synaptic Systems, 266004, 1:500) - Tested reactivity: mouse. KD/KO Validated

Mouse anti-GAD67 (Millipore Sigma, mab5406, 1:500) - Tested reactivity: mouse. This Anti-GAD67 Antibody, clone 1G10.2 is validated for use in IH, IH(P), WB for the detection of GAD67.

Rabbit anti-eIF4H (Cell signaling, 3469T, 1:250) - Reactive with mouse, application key: IHC. For KD validation see paper 'The DEAD-box helicase eIF4A1/2 acts as RNA chaperone during mitotic exit enabling chromatin decondensation'.

Rabbit anti-eEF2 (Proteintech, 14491-1-AP1, 1:500) - Tested reactivity: mouse. KD/KO Validated

Rabbit anti-RPL6 (Novus Biological, NBP2-20216, 1:500) - Reactivity: mouse, Application: IHC. For KD validation see paper 'RPL6 Interacts with HMGCS1 to Stabilize HIF-1α by Promoting Cholesterol Production in Hepatocellular Carcinoma'.

Rabbit anti-RPS7 (Proteintech, 14796-1-AP, 1:500) - Tested reactivity: mouse. KD/KO Validated

Rabbit anti-Phospho-eIF2α (Ser51) (Cell signaling, 3398T, 1:250) - Reactive with mouse, application key: IHC For validation example see paper 'Integrated stress response of vertebrates is regulated by four eIF2α kinases'.

Rabbit anti-total eIF2α (Cell signaling , 5324T, 1:500) - Reactive with mouse, application key: IHC. Used in all paper together with phospho specific antibody above.

Eukaryotic cell lines

Policy information about cell lines and Sex and Gender in Research

Cell line source(s)	Primary cortical neuron cultures were prepared from ~E18 C57BL/6 embryos. HEK293FT (Invitrogen R70007).
Authentication	The identity of HEK293FT was verified by the supplier (Invitrogen). Primary neurons identification was performed based on expression of post-mitotic neuronal markers and on their characteristic morphology.
Mycoplasma contamination	Cell lines were not tested for mycoplasma contamination.
Commonly misidentified lines (See ICLAC register)	None.

Animals and other research organisms

Policy information about studies involving animals; ARRIVE guidelines recommended for reporting animal research, and Sex and Gender in Research

Laboratory animals	C57BL/6 mice were group-housed on a 12-hr light-dark cycle and fed a standard rodent chow diet. Experiments were collected at postnatal day 25.
Wild animals	N/A
Reporting on sex	Only male mice were used in the study to remove sex-driven gene expression variability.
Field-collected samples	N/A
Ethics oversight	All experimental protocols were approved by the Scripps Research Institute Institutional Animal Care and Use Committee (IACUC) and were in accordance with the guidelines from the NIH.

Note that full information on the approval of the study protocol must also be provided in the manuscript.

Plants

Seed stocks	N/A
Novel plant genotypes	N/A
Authentication	N/A
